# The novel role of LOC344887 in the enhancement of hepatocellular carcinoma progression via modulation of SHP1-regulated STAT3/HMGA2 signaling axis

**DOI:** 10.7150/ijbs.99683

**Published:** 2024-12-02

**Authors:** Yang-Hsiang Lin, Hsiang‑Cheng Chi, Meng-Han Wu, Chia-Jung Liao, Cheng-Yi Chen, Po-Shuan Huang, Wei-Chieh Huang, Yi-Wen Wang, Tzu-Kang Lin, Ming-Wei Lai, Chau-Ting Yeh, Kwang-Huei Lin

**Affiliations:** 1 Liver Research Center, Chang Gung Memorial Hospital, Linkou, Taoyuan, Taiwan.; 2 Graduate Institute of Biomedical Sciences, College of Medicine, Chang Gung University, Taoyuan, Taiwan.; 3 Institute of Biochemistry and Molecular Biology, China Medical University, Taichung, Taiwan.; 4 Chinese Medicine Research Center, China Medical University, Taichung, Taiwan.; 5 Department of Cell Biology and Anatomy, College of Medicine, National Cheng Kung University, Tainan, Taiwan.; 6 Graduate Institute of Integrated Medicine, China Medical University, Taichung, Taiwan.; 7 School of Nursing, College of Medicine, Chang Gung University, Taoyuan, Taiwan.; 8 Neurosurgery, School of Medicine, College of Medicine, Fu Jen Catholic University, New Taipei City, Taiwan.; 9 Neurosurgery, Department of Surgery, Fu Jen Catholic University Hospital, New Taipei City, Taiwan.; 10 Division of Pediatric Gastroenterology, Department of Pediatrics, Chang Gung Memorial Hospital Linkou Main Branch, Taoyuan, Taiwan.; 11 Institute of stem cell and translational cancer research, Chang Gung Memorial Hospital, Linkou, Taoyuan, Taiwan.; 12 Research Center for Chinese Herbal Medicine, College of Human Ecology, Chang Gung University of Science and Technology, Taoyuan, Taiwan.

**Keywords:** Hepatocellular carcinoma, LOC344887, Cell motility, Cell signaling, HMGA2, STAT3, SHP-1

## Abstract

Pseudogene-derived long non-coding RNAs (lncRNAs) have become crucial regulators in cancer progression. Extensive research highlights the pivotal role of signal transducer and activator of transcription 3 (STAT3) in promoting hepatocellular carcinoma (HCC) progression. As a result, targeting aberrant STAT3 activation presents a promising therapeutic strategy for HCC. Our study aims to identify the key pseudogene-derived lncRNA involved in modulating STAT3 activation and driving HCC progression. Our study is the first to identify a significant upregulation of LOC344887, a pseudogene-derived lncRNA, in HCC tissues. Elevated LOC344887 levels correlated with poor overall survival (OS) and recurrence-free survival (RFS), highlighting its potential as a biomarker for HCC. The rapid amplification of cDNA ends (RACE) and RT-PCR experiments revealed the expression of a novel LOC344887 transcript, named LOC344887-v2, alongside the annotated RefSeq transcript NR_151491 (LOC344887-v1) in both HCC tissues and hepatoma cell lines. Functional assays demonstrated that LOC344887 enhances cellular migration and invasion, with its variant LOC344887-v2 exhibiting a more pronounced effect. Further, LOC344887 mechanistically regulates STAT3 phosphorylation at tyrosine 705, which is crucial for maintaining STAT3 activation in HCC. Our findings unravel that LOC344887 not only physically interacts with p-STAT3 but also prevents its dephosphorylation by src homology region 2 domain-containing phosphatase 1 (SHP-1), thereby sustaining oncogenic signaling. In addition, we identified HMGA2 as a target of the LOC344887/SHP-1/STAT3 axis, with higher HMGA2 expression correlating with poorer prognosis in HCC patients. The ability of LOC344887 to regulate HMGA2 through direct binding of STAT3 to its promoter underlines its role in HCC progression. Collectively, these findings elucidate a novel oncogenic role of LOC344887 in HCC and suggest that targeting this lncRNA and its associated pathways may provide novel therapeutic strategies for improving patient outcomes in HCC.

## Introduction

Less than 2% of the human genome consists of protein-coding genes, while the majority are non-coding, including microRNAs (miRNA) and pseudogene-derived long non-coding RNAs (lncRNA) 1, 2. These non-coding genes are reported to regulate diverse cellular processes such as cell growth, metabolism, motility, and drug resistance 3, 4. Pseudogenes, in particular, can be transcribed into RNA molecules that modulate gene expression by sponging miRNA or interacting with RNA-binding proteins 5, 6. Pseudogene-derived lncRNAs have emerged as significant regulators of various biological processes, including tumorigenesis, metastasis, and therapeutic response during cancer progression, including in hepatocellular carcinoma (HCC) 7. Proliferating cell nuclear antigen pseudogene 1 (PCNAP1) as one of the notable lncRNAs in HCC, for instance, is shown to modulate hepatitis B virus (HBV) replication, enhance tumor growth 8, while lipoprotein(a) like 2 (LPAL2) is reported to suppress tumor growth and metastasis in HCC by modulating the expression of matrix metalloproteinase 9 (MMP9) 9. Higher expression of oncogene PCNAP1 and downregulation of tumor suppressor LPAL2 are correlated with poor prognosis in HCC patients, underscoring the potential of pseudogene-derived lncRNAs to serve as prognostic indicators and therapeutic targets in HCC 8, 9.

The role of lncRNAs in regulating signaling pathways is also critical for understanding their contributions to HCC. For instance, lncRNA HULC (highly upregulated in liver cancer) has been implicated in promoting HCC via activation of AKT/PI3K/mTOR signaling pathway 10. Similarly, the pseudogene-derived lncRNA pituitary tumor-transforming 3 pseudogene (PTTG3P) is upregulated in HCC tissues 11. Knockdown of PTTG3P inhibits cell growth and motility both *in vitro* and *in vivo*. PTTG3P directly interacts with miR-383, resulting in the upregulation of cyclin D1 and poly ADP-ribose polymerase 2 (PARP2), while also regulating the PI3K/AKT pathway through the PTTG3P/miR-383/cyclin D1/PARP2 axis. These findings highlight the regulatory roles of lncRNAs in cancer signaling networks. Elevated levels of signal transducer and activator of transcription 3 (STAT3) play a pivotal role in persistent activation of key cancer hallmarks such as metastasis, tumor formation, drug resistance, and cancer stemness 12 and are linked to poor prognosis 12. Src homology 2 (SH2) domain-containing protein tyrosine phosphatase 1 (SHP-1) acts as a negative regulator by inhibiting STAT3 phosphorylation, suppressing its sustained activation 13. Since this regulatory mechanism is frequently disrupted in various cancers, unraveling the processes by which the SHP-1/STAT3 pathway drives metastasis can be essential for improving survival outcomes.

Earlier investigations have highlighted the high expression of a pseudogene-derived lncRNA, mRNA like redox sensor 2, pseudogene (NMRAL2P/LOC344887), in gallbladder and non-small cell lung cancer 14, 15. Furthermore, LOC344887 is identified as a target gene of nuclear factor erythroid 2-related factor 2 (NRF2), with implications in pulmonary fibrosis 16. Despite these findings, the functional role of LOC344887-mediated SHP-1/STAT3 signaling and its clinical relevance in HCC remain understudied. In this current study, we aim to elucidate the biological effects and molecular mechanisms of LOC344887 in HCC. Through comprehensive profiling analyses and a series of *in vitro* and *in vivo* experiments, we identified LOC344887 as a critical regulator of STAT3 activation by inhibiting SHP-1-mediated dephosphorylation of STAT3. Additionally, the LOC344887/STAT3 axis drives the upregulation of high mobility group AT-hook 2 (HMGA2), a key factor in promoting cell motility. Our findings offer valuable insights into the role of LOC344887 in modulating the SHP-1/STAT3/HMGA2 signaling pathway, positioning it as a key regulator and a promising therapeutic target in the metastatic progression of HCC.

## Materials and Methods

### HCC specimens

Paired HCC samples (n = 158) were obtained from the Taiwan Liver Cancer Network (TLCN) and were subsequently subjected to qRT-PCR and immunoblotting analysis. The tissue array, procured from TissueArray.com LLC (Derwood, MD, USA, BC03117a), underwent *in situ* hybridization and immunohistochemistry staining. All assays were conducted in accordance with the approval granted by the Medical Ethics and Human Clinical Trial Committee at Chang Gung Memorial Hospital (IRB: 201901859B0).

### Statistical analysis

All data are expressed as means ± SD from three independent experiments. Statistical analysis was performed using IBM SPSS software (SPSS Inc., Chicago, IL, USA, version 20). The Student's t-test or Mann-Whitney test was employed to compare two groups, while one-way ANOVA followed by the Tukey post-hoc test was used for two or more groups. Kaplan-Meier methods were utilized to analyze survival outcomes, specifically overall survival and recurrence-free survival, with death as an event, and statistical significance was determined using the log-rank test. P values less than 0.05 were considered statistically significant.

The detailed descriptions of methods and materials are described in the Supplemental information.

## Results

### Identification of the pseudogene-derived lncRNA LOC344887 as a prognostic factor in HCC

**Figure 1:**
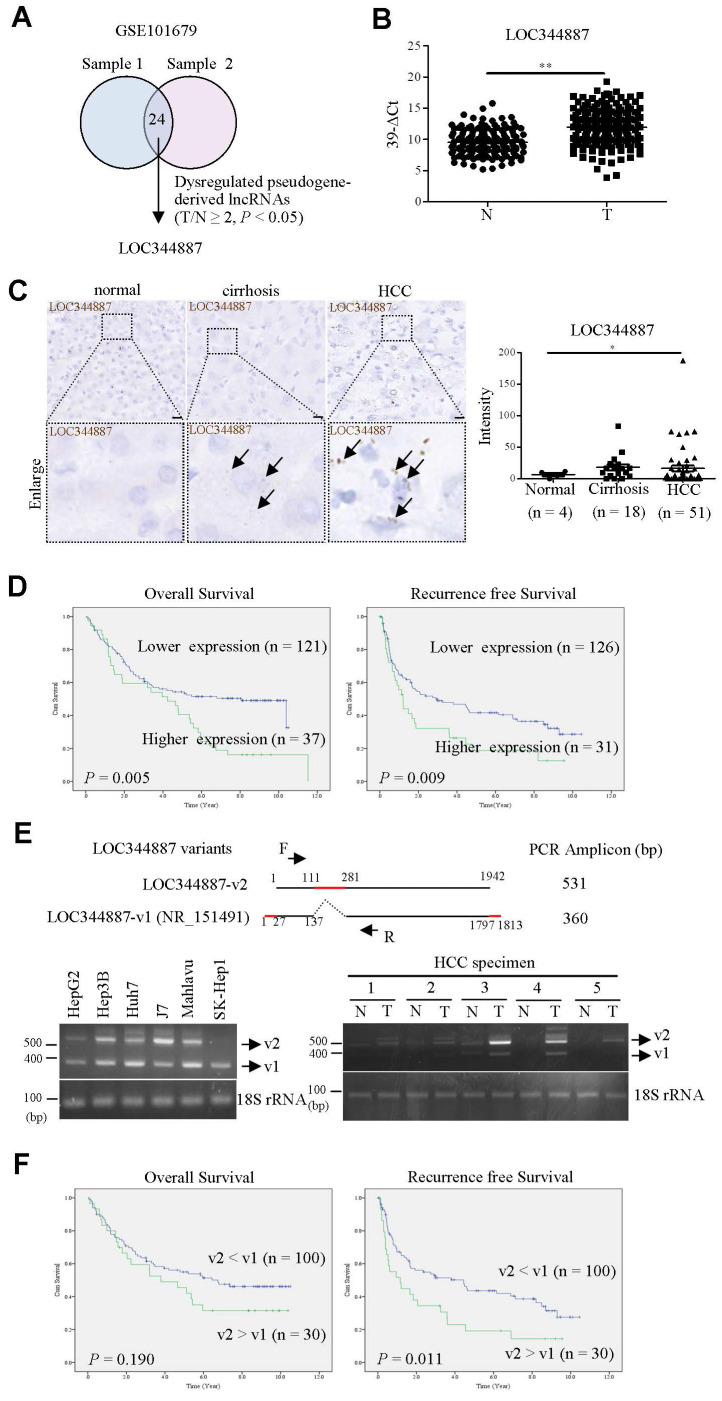
** lncRNA LOC344887 is highly expressed in HCC and correlated to survival outcomes.** (A) Venn diagram was utilized to illustrate significantly differentially expressed pseudogene-derived lncRNAs from two sets of paired HCC specimens containing tumor and adjacent normal tissues (GSE101679) 17. LOC344887 was the pseudogene-derived lncRNA most significantly upregulated in HCC tissues out of the total of 24 identified pseudogene-derived lncRNAs, which were differentially expressed by at least 2-fold in HCC as compared to adjacent normal tissues (P < 0.05). (B) Association of LOC34487 to tumor progression was determined by measuring the expression levels of LOC344887 in HCC tumors (n = 158) as compared to adjacent normal tissues (n = 158) using qRT-PCR. The results are presented as 39-∆Ct values. N: adjacent normal tissue; T: HCC tumors. Statistical significance was analyzed using the Mann-Whitney test. (C) LOC344887 expression was further validated by employing *in situ* hybridization (ISH) on tissue array. The brown signal (indicated by arrowhead) represents the intensity of LOC344887 signals (left panel), which were statistically quantified according to specimens grouped into normal (n = 4), cirrhotic (n = 18), and HCC tissues (n = 51) (right panel). Statistical significance was determined using one-way ANOVA followed by Tukey's post-hoc test. Scale bar, 100 μm. (D) Higher expression of LOC344887 was correlated to lower survival outcomes as determined by Kaplan-Meier log-rank analysis. Mean values of LOC344887 were used as the cutoff. (E) A schematic representation illustrates the two LOC344887 variants investigated in this study (upper panel), with the red line indicating the unique sequences for the v1 and v2 transcripts of LOC344887. RT-PCR primers (forward primer: F, reverse primer: R) were designed to specifically detect and distinguish the two LOC344887 transcripts (PCR amplicons for LOC344887-v1 and LOC344887-v2 were 360 bp and 531 bp, respectively). RT-PCR analysis was used to determine the expression of LOC344887 variants (v1 and v2) in six different hepatoma cell lines (lower left panel) and HCC specimens (lower right panel), showing varying expression levels for the two variants. 18S rRNA served as loading controls. The full-length sequences of LOC344887 transcripts are depicted in Supplementary Figure 1. (F) Correlation of the two LOC344887 variants to clinical OS and RFS was examined by RT-PCR analysis, which calculated fold-changes of LOC344887-v2 relative to LOC344887-v1 and categorized into higher expression group (expression level of LOC344887-v2 > LOC344887-v1) and lower expression group (expression level of LOC344887-v2 < LOC344887-v1). The survival outcomes in relation to LOC344887-v1 and v2 in these HCC specimens were then analyzed using the Kaplan-Meier log-rank analysis (*, P < 0.05; **, P < 0.01).

Despite pseudogene-derived lncRNAs have recently been implicated in tipping the control of cancer progression, the role of lncRNA in promoting or suppressing HCC progression remains largely controversial. In current study, microarray profiling analysis on two sets of HCC specimens and their corresponding adjacent normal tissues (GSE101679) 17 was conducted in aim to identify pseudogene-derived lncRNAs pivotal for modulating disease progression of HCC. Our analysis uncovered 24 pseudogene-derived lncRNAs that were significantly upregulated by at least 2-fold in HCC tissues when compared to adjacent normal tissues (Figure 1A). LOC344887 emerged as the most differentially expressed pseudogene-derived lncRNA among all candidates, and was further investigated (highlighted in bold in Table 1). To validate LOC344887 as the prominently dysregulated lncRNA from the profiling analysis, qRT-PCR and *in situ* hybridization (ISH) analyses were conducted. qRT-PCR analysis revealed that LOC344887 was markedly upregulated in HCC specimens when compared to adjacent normal tissues (Figure 1B). In contrast, ISH analysis also demonstrated significantly augmented expression of LOC344887 in HCC but not cirrhosis specimens (Figure 1C). These elevated LOC344887 expressions observed in HCC patients were substantiated by further investigating its clinicopathological implication. Our survival analysis showed that higher expression of LOC344887 was significantly correlated with lower overall survival (OS) and recurrence-free survival (RFS) (Figure 1D). In addition, LOC344887 elicited clinicopathologically significant associations with tumor type, vascular invasion, and pathological stage (Table 2). To corroborate the clinicopathological role of LOC344887 in HCC, we first characterized the LOC344887 transcript expressed in hepatoma cell lines through 5′ and 3′ rapid amplification of cDNA ends (RACE) assays. Intriguingly, a novel LOC344887 variant (designated as v2) was identified from the J7 hepatoma cell line (Supplementary Figure 1). As shown in the upper panel of Figure 1E, LOC344887-v2 was distinguishable from canonical LOC344887 transcript (LOC344887-v1, annotated RefSeq transcript, NR_151491) that had a 170 bp insert (111 to 281 bp) and shorter 5′/3′ ends. To ascertain whether the expression of LOC344887 variants differs in hepatoma cell lines and HCC tissues, RT-PCR was performed on six HCC cell lines and specimen pairs. The results showed that both v1 and v2 variants of LOC344887 were predominantly expressed in HCC cell lines except for SK-Hep1 (Figure 1E, lower left panel). In clinical specimens, LOC344887-v2 elicited higher expression than LOC344887-v1 and was primarily expressed in HCC but not adjacent normal tissues (Figure 1E, lower right panel). Next, clinical implications of the two LOC344887 variants were substantiated by determining correlation of respective expression to clinical survival rates. Our data demonstrated that significantly higher ratios of LOC344887-v2/LOC344887-v1 expression were associated with poorer RFS than patients with lower expression ratios (Figure 1F), suggesting pertinent clinicopathological role of LOC344887 in HCC.

### LOC344887 expression deploys cellular migration and invasion to mediate HCC progression* in vitro* and *in vivo*

**Figure 2:**
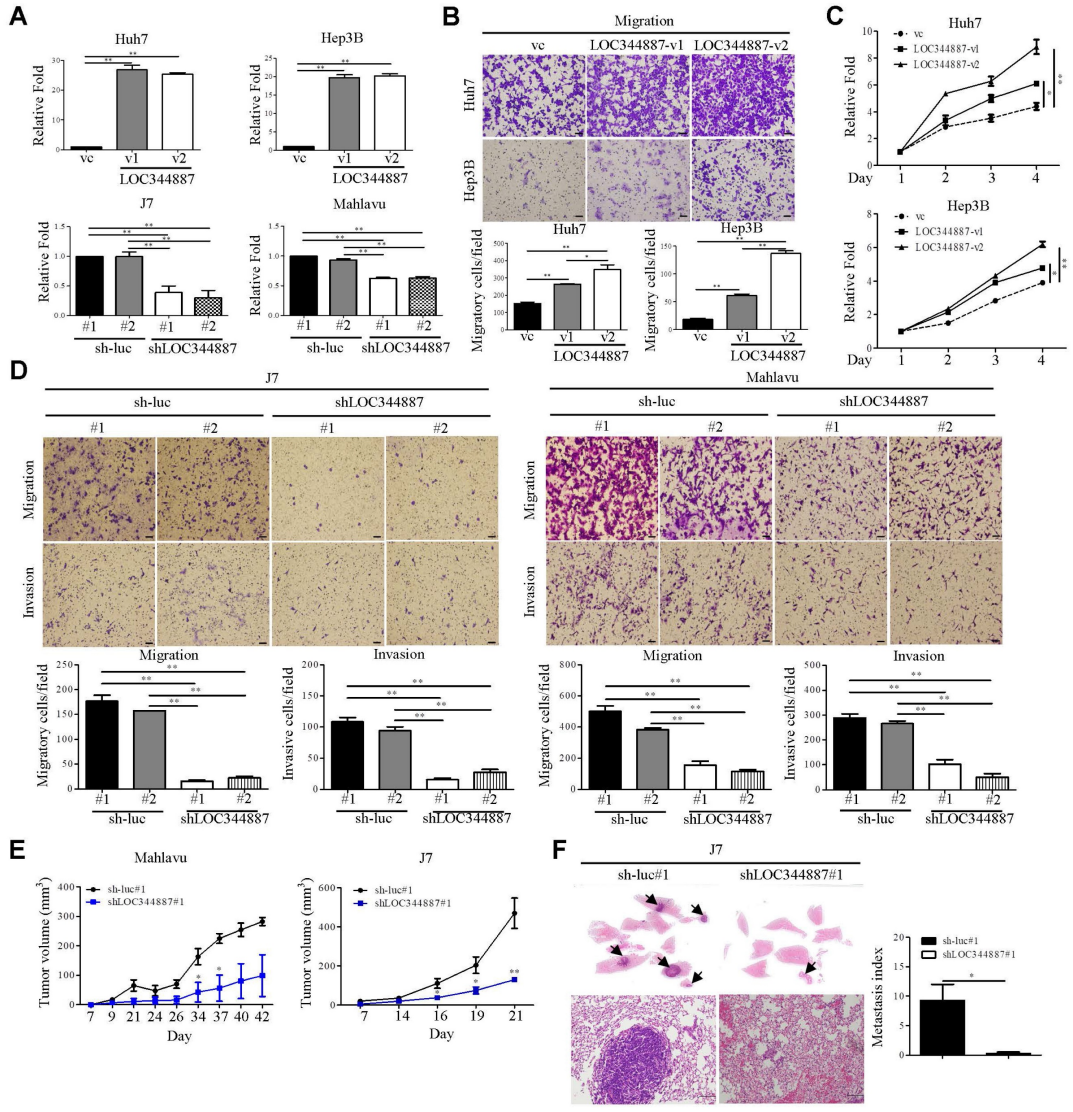
** LOC344887 expression is pertinent to cellular growth and cell motility of HCC *in vitro* and *in vivo*.** (A) Two LOC344887 stable overexpression models (Huh7 and Hep3B) and stable knockdown models (J7 and Mahlavu) were established to assess the role of LOC344887 in HCC progression. Two LOC344887 variants and two shRNAs were employed in each HCC model. Expression levels of LOC344887 in these stable cell lines were determined via qRT-PCR analysis using 18S rRNA as the loading control. vc: vector control. (B) Cell migration assays were performed using stable cell lines, each overexpressing LOC344887 (LOC344887-v1 or LOC344887-v2). Migratory cells were stained with crystal violet, and cell counts were quantified using ImageJ software, showing significantly increased migration ability from cells overexpressing both variants. Scale bar, 100 μm. vc: vector control. (C) Cell growth of HCC impacted by LOC344887 was determined by monitoring cellular growth of LOC344887-overexpressing stable cell lines (LOC344887-v1 and LOC344887-v2) over 3 days the MTT assay. Data were normalized to values of viable cells on day 1 for each group and presented as fold changes. (D) Migration and invasion abilities of HCC cells impacted by LOC344887 knockdown were assessed using control cells (sh-luc#1 or sh-luc#2) and LOC344887-depleted cells (shLOC344887#1 or shLOC344887#2). Crystal violet-stained migratory and invasive cells were both significantly reduced upon LOC344887 knockdown. Scale bar: 100 μm. Statistical significances for *in vitro* assays were derived from one-way ANOVA followed by Tukey's post-hoc test. *, P < 0.05; **, P < 0.01. (E) The role of LOC344887 in *in vivo* tumor growth of HCC was evaluated using xenograft model by inoculating LOC344887 knockdown cells (shLOC344887#1) or control cells (sh-luc#1) subcutaneously into nude mice. (F) Hematoxylin and eosin (H&E) staining of lung tissues from J7-sh-luc and J7-shLOC344887#1 mice (n = 4 per group) was performed to uncover the inhibitory function of LOC34887 knockdown in metastasis. Metastases in lung tissues are indicated by arrowheads, and metastasis index was calculated from relative fold changes in number of metastasized cells. Scale bar, 50 μm. Statistical significances for *in vivo* experiments were obtained using the Mann-Whitney test. *, P < 0.05; **, P < 0.01.

To evaluate whether LOC344887 expression contributes to HCC progression, stable overexpression (Huh7 and Hep3B) and knockdown (J7 and Mahlavu) models of LOC344887 were first established (Figure 2A). The ectopic expression of LOC344887-v1 and LOC344887-v2 variant significantly enhanced cellular migration ability of both Huh7 and Hep3B cells (Figure 2B). Of note, migration ability induced by LOC344887-v2 was significantly more pronounced than that of LOC344887-v1. Similarly, cellular proliferation of both Huh7 and Hep3B models was significantly elevated in cells overexpressing LOC344887-v1 or LOC344887-v2 (Figure 2C). Conversely, lost-of-function assays using LOC344887-depleted J7 and Mahlavu models were employed to validate the greatly enhanced migration and invasion capacities imposed by LOC344887 overexpression. The results showed that LOC344887 knockdown by two LOC344887-specific shRNA led to significantly ablated numbers of migratory and invasive cells when compared to control shRNA (sh-luc) knockdown cells of J7 and Mahlavu (Figure 2D). To extrapolate whether these *in vitro* effects of LOC344887 in modulating cellular migration and invasion could be recapitulated in preclinical settings, *in vivo* function of LOC344887 was assessed by tumor formation and tumor cell metastasis assays in nude mice and severe combined immunodeficient (SCID) mice, respectively. As shown in Figure 2E, LOC344887-depleted Mahlavu and J7 tumors grew at significantly reduced rate than that of sh-luc control tumors. In addition, lung metastasis of J7 xenograft model elicited near ablation in metastasized cancer cells upon LOC344887 depletion (Figure 2F). These observations hence implicated a potent role of LOC344887 in coordinating HCC tumorigenesis.

### LOC344887 expression is pertinent to regulation of STAT3 phosphorylation at Tyrosine 705

**Figure 3:**
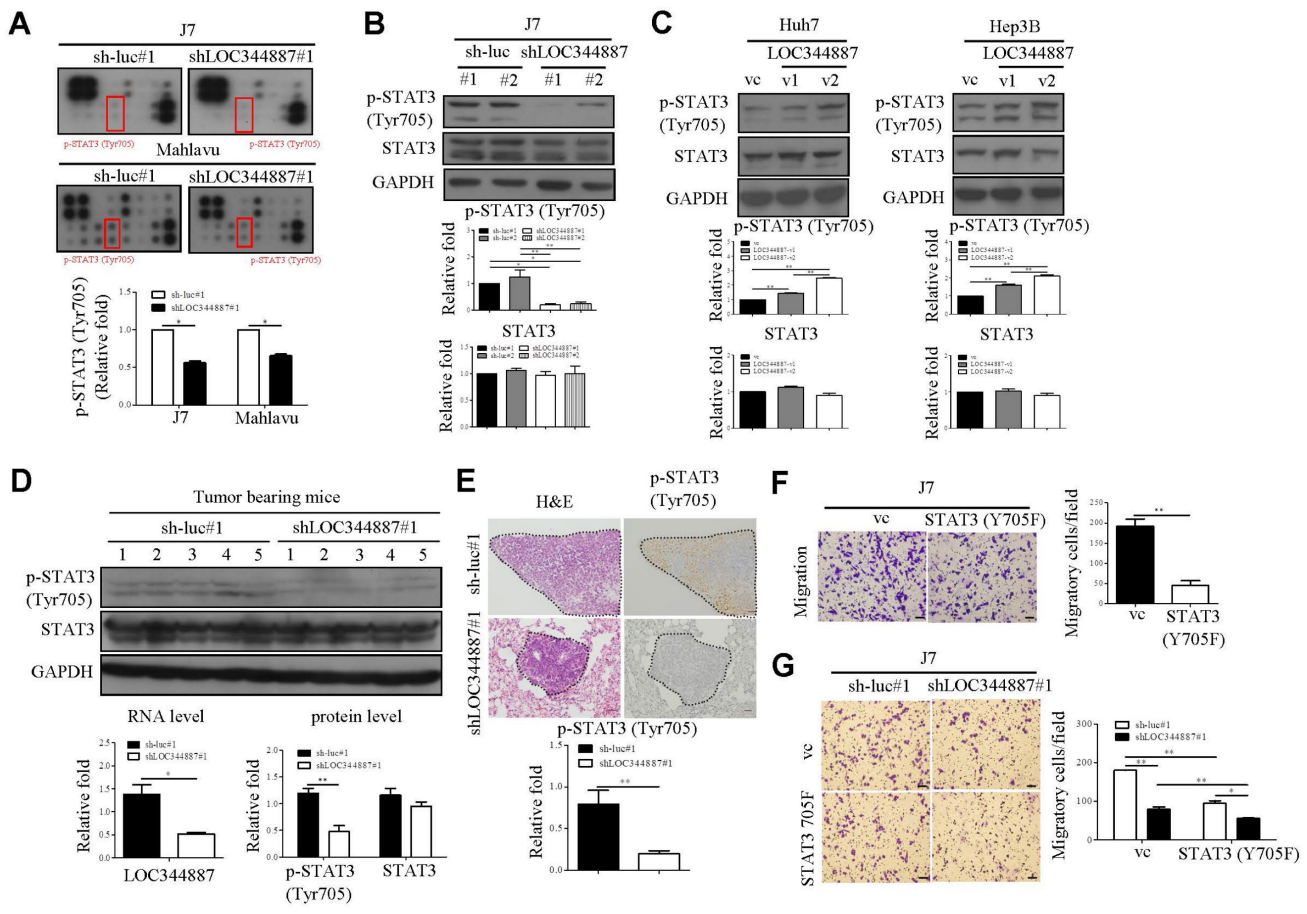
** LOC344887 mediates STAT3 activation to promote HCC migration.** (A) A human JAK/STAT pathway phosphorylation kit (RayBiotech) was utilized to assess the signaling pathway impacted by LOC344887 knockdown (shLOC344887#1) in J7 and Mahlavu cells by comparison to control cell lines (sh-luc#1). The expression levels of p-STAT3 (Tyr705) marked by red rectangular boxes were quantified and presented as average fold changes using Student's t-test. The phosphorylation level of STAT3 (Tyr705) in (B) J7 LOC344887 stable knockdown cell line and (C) Huh7, Hep3B LOC344887-v1, -v2 stable overexpression cell lines were determined by western blot analysis, showing prominent association of LOC344887 with STAT3 signaling activation. GAPDH was used as a protein loading control. The values in each lane represent the quantified results relative to the control group (sh-luc or vc, n = 3). (D) Xenografts from nude mice transplanted with sh-luc#1 or shLOC344887#1 cells were collected for STAT3 activation analysis (same xenografts as tumor formation assay in Figure 2E). The expression levels of p-STAT3 (Tyr705) and total STAT3 were quantified and presented as average fold changes. LOC344887 expression levels in these xenografts were measured by qRT-PCR using 18S rRNA as the RNA loading control. Statistical significance was determined using Student's t-test. (E) Signal intensities of p-STAT3 in metastasized tumors from SCID mice transplanted with sh-luc#1 or shLOC344887#1 cells (same lung metastases from Figure 2F) were measured using IHC staining. A dotted line indicates the tumor region and p-STAT3 expression levels were quantified and presented as average fold change. The scale bar represents 100 μm. (F) Effects of STAT3 phosphorylation on cellular migration were assessed using J7 cells overexpressed with a STAT3 Tyr705 dominant negative (DN, Y705F) in Transwell assays. Migratory cells stained with crystal violet were quantified using ImageJ software (scale bar represents 100 μm). Statistical significances were obtained using Student's t-test. (G) The effects of STAT3-DN (Y705F) on HCC migration were further examined using LOC334487 stable knockdown J7 cells. Statistical significances among knockdown (shLOC344887#1) and control (sh-luc#1) groups with or without Y705F overexpression were determined using one-way ANOVA followed by Tukey's post-hoc test. *, P < 0.05; **, P < 0.01.

To substantiate underlying mechanisms driving LOC344887-mediated oncogenic effects in HCC, microarray profiling using LOC344887-depleted stable J7 cell line was carried out as depicted in Supplementary Figure 2A. Resulting microarray data analysis by KEGG revealed prominently enhanced pathways including pathways in cancer and cytokine-cytokine receptor interaction when LOC344887 was depleted (Supplementary Figure 2B, 2C). The JAK-STAT pathway, included in KEGG pathways in cancer, is associated with the oncogenesis of various cancer types, including HCC 18, 19. To confirm the signaling mechanisms modulated by LOC344887, a profiling analysis using a JAK/STAT phosphorylation array was conducted. As shown in Figure 3A (red rectangular box), the phosphorylation levels of STAT3 at Tyrosine 705 (Tyr705) was significantly reduced as compared to those of sh-luc controls in J7 cells. Similar reductions in p-STAT3 (Tyr705) by LOC344887 knockdown were observed in Mahlavu cells, suggesting potential association of LOC344887 to STAT3 signaling in HCC (Figure 3A). The pivotal identification of STAT3 phosphorylation level from LOC344887-depleted phosphorylation array was confirmed by assessing the expression levels of p-STAT3 (Tyr705) in LOC344887 stable cell lines through western blot analysis. Consistently, knockdown of LOC344887 resulted in mitigated expression of phosphorylated STAT3 (Tyr705) (Figure 3B), while overexpression of LOC344887-v1 and -v2 significantly augmented p-STAT3 (Tyr705) expression when compared to vector control group (Figure 3C). Notably, LOC34487-v2 elicited significantly more pronounced inductions in STAT3 phosphorylation than LOC344887-v1 transcript, implicating a potential mechanism through which LOC344887 mediated cellular migration and proliferation as observed in Figure 2B and 2C. To ratify LOC344887-mediated STAT3 phosphorylation *in vivo*, xenografts from J7 tumor-bearing models were evaluated for their expression of p-STAT3. Our data demonstrated that expression levels of p-STAT3 in the LOC344887 knockdown xenografts were significantly lower than those of sh-luc control xenografts (Figure 3D). In addition, IHC staining results also elicited significantly attenuated phosphorylation level of p-STAT3 in the LOC344887-depleted xenografts (Figure 3E). To corroborate the influence of STAT3 phosphorylation on cell motility in HCC, a dominant-negative STAT3 tyrosine 705 mutant (STAT3-Y705F) was employed. Our results showed that overexpression of the STAT3-Y705F mutant largely alleviated the number of migratory J7 cells as compared to control group (Figure 3F). Moreover, overexpression of STAT3-Y705F not only reduced cell mobility in control cells, but also further mitigated the reduced cell motility lowered by LOC344887 knockdown (Figure 3G). These findings suggested that LOC344887 contributed to HCC migration via STAT3 signaling activation.

### LOC344887 variant 2 is a more potent regulator in STAT3 signaling and HCC migration

**Figure 4:**
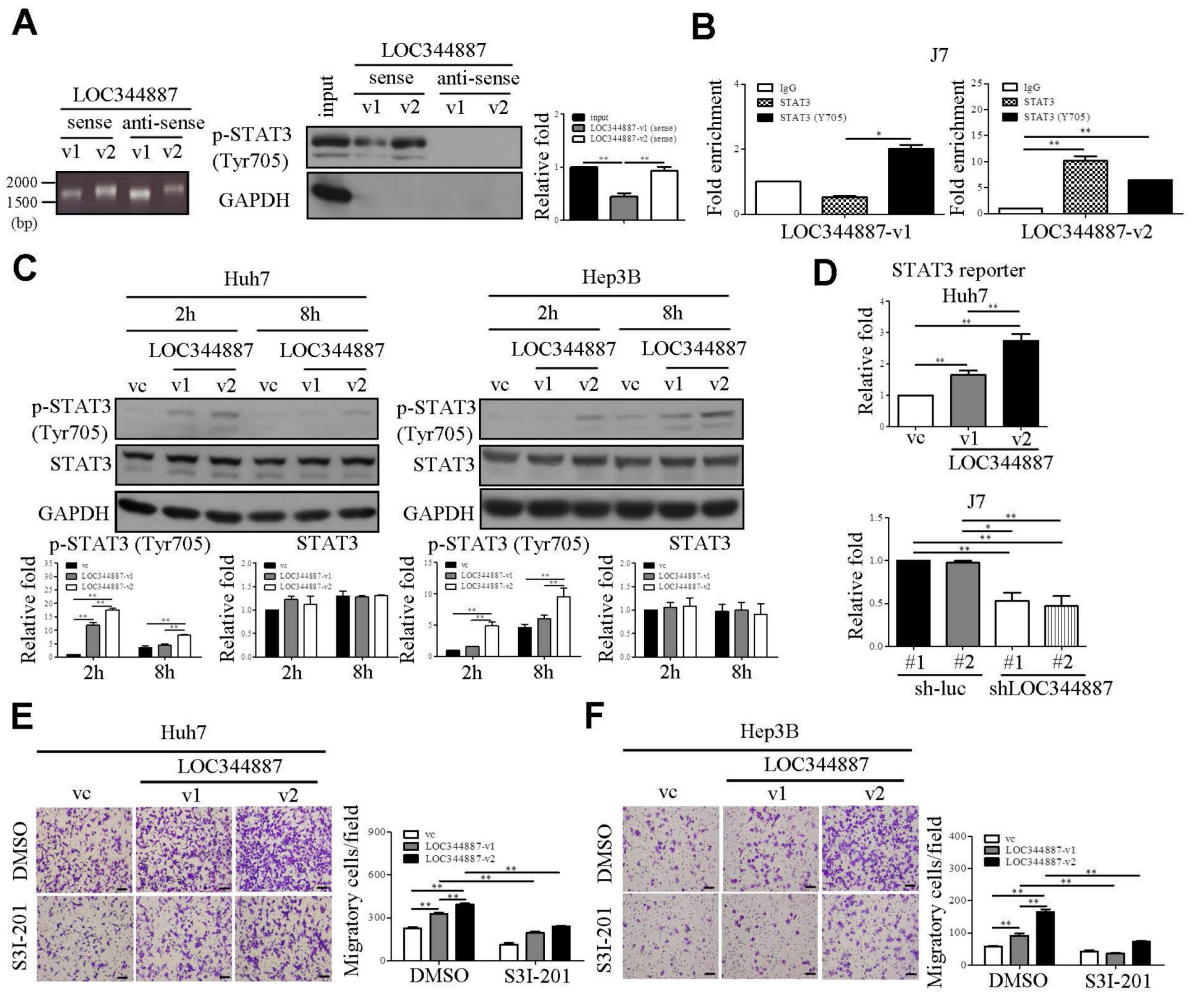
** LOC344887 interacts with STAT3 to promote phosphorylation of STAT3 and HCC migration.** (A) The RNA pull-down assay using biotinylated LOC344887-v1 (sense) and LOC344887-v2 (sense) elicited interaction with endogenous pSTAT3 (Tyr705) as confirmed by western blot analysis, in which GAPDH was used as negative control (right panel). The quantitative results of p-STAT3 (n = 3) were normalized to the input for both sense strands of v1 and v2 transcripts. The integrity of the sense and antisense strands of LOC344887 transcripts was confirmed using RNA agarose gel electrophoresis (left panel). (B) RNA immunoprecipitation (RIP) assays using antibodies against STAT3 and p-STAT3 (Tyr705) were conducted to investigate physical association of LOC344887-v1 (v1) and LOC344887-v2 (v2) with STAT3 or phosphor-STAT3 in J7 cells, showing significantly enriched LOC344887-v1 and LOC344887-v2 in p-STAT3 (Tyr705) and STAT3 immunoprecipitates. Fold enrichment values were normalized to IgG. (C) At 2 and 8 hours post-transient transfection with vector control, LOC344887-v1 and LOC344887-v2 in Huh7 and Hep3B cell lines, p-STAT3 (Tyr705), STAT3 and GAPDH levels were assessed by western blot. The quantitative results of p-STAT3 or STAT3 (n = 3) were normalized to that of vc for both v1 and v2 transcripts, showing significant increases in p-STAT3 in cells overexpressing v1 and v2. (D) STAT3 luciferase reporter activity was assessed 48 hours after transfection into Huh7 LOC344887-overexpression or J7 LOC344887-knockdown stable cell lines, showing the ability of LOC344887 in enhancing or reducing p-STAT3 levels, respectively. Fold changes in reporter activities were calculated by normalizing to control groups (vc or sh-luc#1). Huh7 (E) and Hep3B (F) cells overexpressing LOC344887-v1 or LOC344887-v2 were treated with or without STAT3 inhibitor (S3I-201, 100 μM) to facilitate cell motility analysis, which showed specific inhibition in v1- and v2-induced motility. Scale bar: 100 μm. Statistical significances indicated in this figure were obtained from one-way ANOVA followed by Tukey's post-hoc test. *, P < 0.05; **, P < 0.01.

LncRNAs have increasingly been recognized for their roles in cancer progression as numbers of studies have demonstrated the ability of lncRNAs in binding with phospho-proteins to prevent dephosphorylation events, thereby promoting cancer development 20, 21. Hence, we next investigated whether LOC344887 could physically interact with STAT3 or p-STAT3 (Tyr705) by conducting RNA pull-down and RNA immunoprecipitation (RIP) assays. Our results demonstrated that sense but not anti-sense strand of both LOC344887 transcripts (v1 and v2) interacted with p-STAT3 (Tyr705), while LOC344887-v2 elicited a stronger binding affinity to p-STAT3 (Tyr705) than LOC344887-v1 in J7 cells (Figure 4A). Consistently, RIP data showed significantly elevated binding of v2 to both total STAT3 and p-STAT3 (Tyr705), whereas stronger binding to p-STAT3 than total STAT3 was observed in v1-overexpresssing cells (Figure 4B), implicating variant-specific modulation of STAT3 signaling activation by LOC344887 in HCC. To gain further insights into the differential effects of LOC344887-v1 and -v2 on STAT3 phosphorylation, a time-course experiment was conducted to examine changes in p-STAT3 (Tyr705) regulated by LOC344887 variants. Two hours after transient transfection of LOC344887 variants into Huh7 and Hep3B cells, both LOC344887-v1 and LOC344887-v2 rapidly induced STAT3 phosphorylation, with LOC344887-v2 showing a significantly stronger induction than that of LOC344887-v1 (Figure 4C). Of note, similar p-STAT3 induction patterns were observed after 8 hours of transfection that showed consistent STAT3 signaling activation by LOC344887-v1, with LOC344887-v2 manifesting significantly higher level of p-STAT3 in both Huh7 and Hep3B cells (Figure 4C). Further, the potent role of LOC344887 in modulating STAT3 signaling activation was substantiated by employing STAT3 reporter assay. In the Huh7 LOC344887-overexpressing cells, reporter activity of STAT3 was not only significantly increased by LOC344887-v1, but also further augmented when LOC344887-v2 was overexpressed (Figure 4D). Conversely, knockdown of LOC344887 resulted in significantly reduced STAT3 reporter activity, exhibiting consistent patterns of STAT3 signaling activation. These findings were corroborated by assessing cell motility of LOC344887-overexpressing Huh7 and Hep3B stable cell lines that were subjected to treatment with a STAT3-specific inhibitor, S3I-201. The results revealed that treatment of S3I-201 in significantly mitigated the elevated cell motilities of LOC344887-v1 and LOC344887-v2-overexpressing Huh7 cells (Figure 4E). Similarly, the largely increased numbers of migratory cells in both variants of LOC344887-overexpressing Hep3B stable cell lines were significantly reduced when S3I-201 was administered (Figure 4F). In line with observations from Figure 3, these findings accentuated the function of LOC344887 in mediating cell motility of HCC via direct interaction and activation of STAT3 signaling.

### LOC344887 modulates SHP-1-mediated STAT3 dephosphorylation

**Figure 5:**
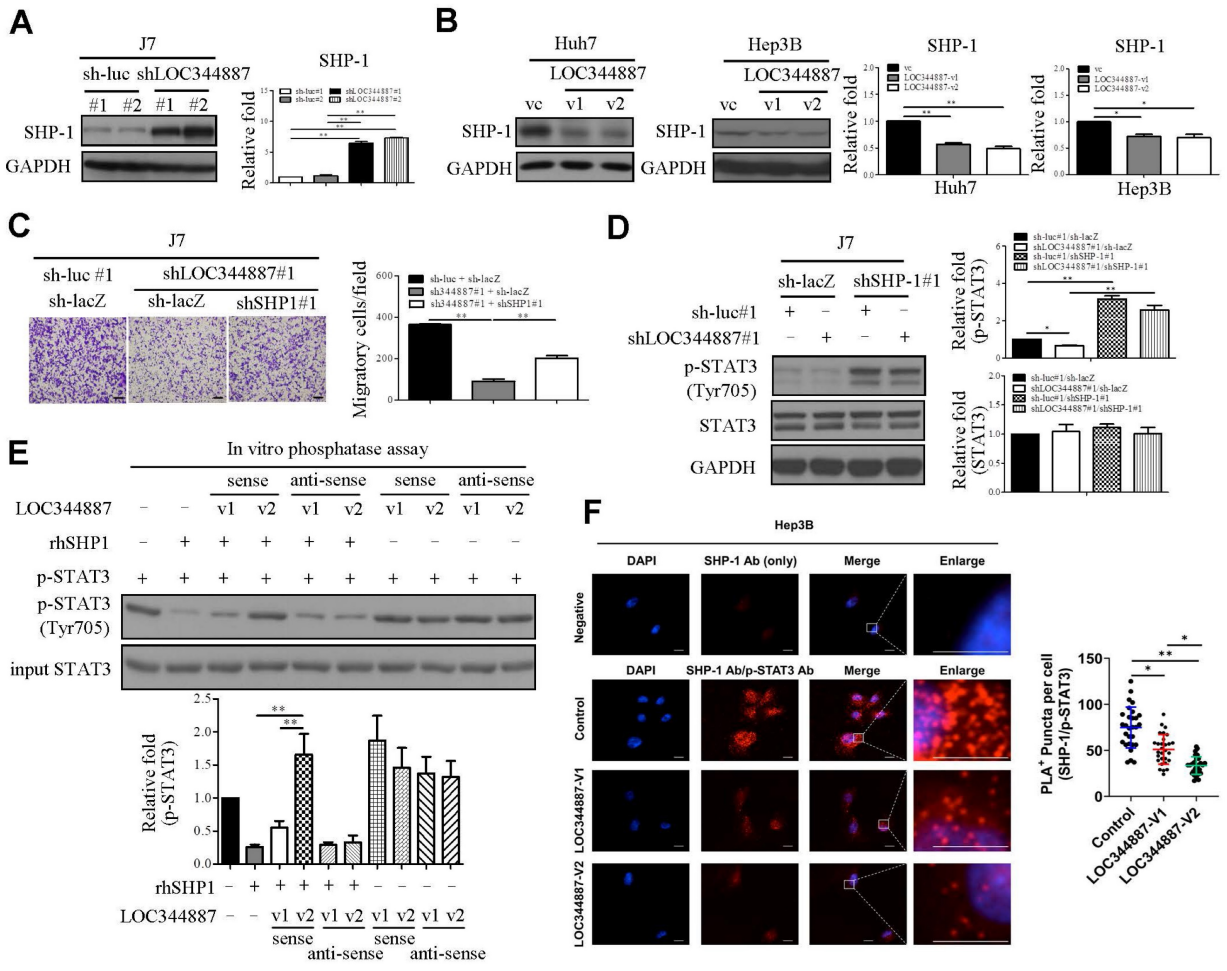
** LOC344887 mediates STAT3 phosphorylation by regulating SHP-1 expression and its interaction with STAT3.** SHP-1 immunoblotting was performed to assess SHP-1 expression in (A) LOC344887 stable knockdown J7 cell lines and (B) LOC344887-v1/-v2 stable overexpression Huh7 and Hep3B cell lines, showing significantly enhanced and reduced expression of SHP-1, respectively. Quantitative results of immunoblots analysis (n = 3) were presented relative to the control groups using GAPDH as loading control. (C) Effects of LOC344887 and SHP-1 expression on cell migration were assessed by knocking down LOC344887 and/or SHP-1 in J7 cells transduced with shLOC344887#1 and/or SHP-1 (shSHP-1#1), showing knockdown of SHP-1 significantly reverted the reduced cell motility by shLOC344887. Sh-luc#1 and sh-lacZ served as control groups. The scale bar is 100 μm. (D) The expression levels of p-STAT3 in LOC344887 and/or SHP-1 in knockdown J7 cells were determined to evaluate the combined effects of LOC344887 and SHP-1 on STAT3 phosphorylation at Tyr705. Quantitative results of immunoblots (n = 3) were obtained by normalizing p-STAT3 or STAT3 to the control groups (lane 1: sh-luc#1/sh-lacZ) as GAPDH served as an internal control. (E) *In vitro* phosphatase assays using recombinant human SHP1 (rhSHP1) incubated in the presence or absence of biotinylated LOC344887 RNA variants (v1 and v2) to allow investigation for the roles of LOC344887-v1/-v2 and SHP1 in mediating STAT3 phosphorylation. The results indicated differential influences from LOC344887-v1 and -v2 sense strands (lane 3, 4) on preventing dephosphorylation effects from rhSHP1. The quantitative results (n = 3) were presented relative to the control group (lane 1). (F) The differential influences of LOC344887-v1 and -v2 on the interaction between SHP-1 and p-STAT3 (Tyr705) was assessed via Duolink proximity ligation assay (PLA) using control-, LOC344887-v1- or LOC344887-v2-overexpressing J7 cells. The results showed a significantly lower association between SHP1/p-STAT3 and LOC344887-v2-overexpression, suggesting the lowest dephosphorylation rate takes place. The proximity of SHP1/p-STAT3 was defined as less than 40 nm and visualized by discrete red fluorescent puncta under fluorescence microscopy. Nuclei were counterstained with DAPI. Scale bars: 10 μm. *, P < 0.05; **, P < 0.01.

To delineate underlying mechanism by which LOC344887 utilizes to maintain STAT3 phosphorylation, members of the protein tyrosine phosphatase family such as SHP-1 known to reduce STAT3 activity 22 were next investigated using stable LOC344887 knockdown and overexpression cell lines. Immunoblotting analyses revealed that SHP-1 protein levels were significantly increased in LOC344887-depleted J7 cells (Figure 5A), whereas overexpressing LOC344887-v1 and -v2 conversely reduced SHP-1 expression in Huh7 and Hep3B cells (Figure 5B). To investigate the mechanistic interplay between SHP-1 and LOC344887/STAT3-mediated oncogenic effects, the role of SHP-1 in cell motility of HCC was first assessed. In line with previous report 23, knockdown of SHP-1 in J7 cell lines resulted in significantly increased cell migration (Supplementary Figure 3A), while overexpressing SHP-1 led to reduced cell migration (Supplementary Figure 3B). In J7 cells depleted of LOC344887, the mitigated cell motility was significantly restored upon SHP-1 knockdown (Figure 5C). Notably, the elevated cell mobility of HCC cooperatively modulated by LOC344887 and SHP1 was attributed to significantly activated STAT3 phosphorylation by SHP-1 knockdown (Figure 5D), as the reduced p-STAT3 by LOC344887 knockdown was greatly ameliorated under SHP-1 knockdown (Figure 5D, lane 2 vs. lane 4). These observations suggested that the phosphatase SHP-1 could specifically dephosphorylate LOC344887-mediated STAT3 signaling to regulate cell motility of HCC. Furthermore, these findings were corroborated by *in vitro* phosphatase assay, in which recombinant human SHP-1 (rhSHP-1) and LOC344887 variants were employed in STAT3 dephosphorylation reactions. The data demonstrated that the phosphorylation level of STAT3 was not mitigated under the presence of LOC344887-v2 sense strand (Figure 5E) due to the stronger association between LOC344887-v2 and p-STAT3 (Figures 4A and 4B), while rhSHP-1 significantly lowered p-STAT3 when LOC344887-v1 sense strand was included in the reaction. Moreover, the molecular interplay among LOC344887-v1/-v2, STAT3 and SHP-1 was corroborated by overexpressing LOC344887-v1 or -v2 and assessing for the level of interaction between SHP-1 and p-STAT3 using duolink proximity assay. Our results uncovered that overexpressing LOC344887-v2 manifested a significantly augmented suppression in the interaction between SHP-1 and p-STAT3 than that of LOC344887-v1 (Figure 5F). Together, these findings suggested that the more robust STAT3 phosphorylation supported by LOC344887-v2 could be attributed to its stronger association with p-STAT3 (Figures 4A and 4B) to prevent SHP-1/p-STAT3 interaction, leading to elevation in cellular migration of HCC.

### HMGA2 is a prime target of LOC344887/SHP-1/STAT3/ signaling axis in HCC

**Figure 6:**
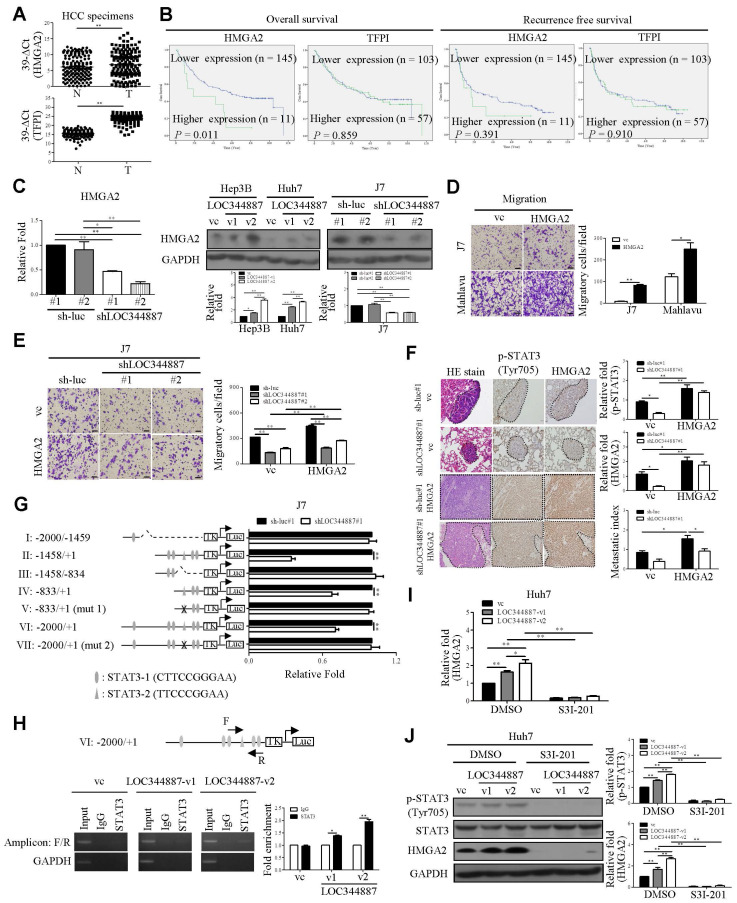
** HMGA2 is upregulated by LOC344887/STAT3 axis and is pivotal to LOC344887/STAT3-mediated cell motility.** (A) The mRNA levels of HMGA2 and TFPI in HCC specimens were measured using qRT-PCR using GAPDH as the loading control. Results were expressed as 39-∆Ct values, showing significantly increased expression of HMGA2 and TFPI in HCC. Statistical significances were determined using the Mann-Whitney test. (B) OS and RFS for HMGA2 and TFPI in HCC were analyzed using Kaplan-Meier, showing only HMGA2 expression was correlated to poor prognosis. The mean values of HMGA2 and TFPI served as the cutoff points for each analysis. (C) mRNA and protein expression levels of HMGA2 in LOC344887 stable knockdown and overexpression cell lines were determined, showing significantly lowered HMGA2 expression in LOC344887-depleted J7 cells and enhanced HMGA2 expression in LOC344887-v1/-v2-overexpressing cell lines. Statistical significances (n = 3) were derived from one-way ANOVA followed by Tukey's post-hoc test. (D) The effect of HMGA2 on HCC cell migration was measured in J7 and Mahlavu cells by Transwell assay, showing significantly increased cell motility when HMGA2 was overexpressed. The indicated scale bar was 100 μm. Statistical significance was determined using Student's t-test. (E) Collective impacts of LOC344887 and HMGA2 on cell migration were evaluated by overexpressing HMGA2 in LOC344887 knockdown J7 cells, showing significantly reverted cell migration when HMGA2 was overexpressed. Statistical significances were derived from one-way ANOVA followed by Tukey's post-hoc test. (F) Hematoxylin and eosin (H&E) staining was performed on lung tissue from the following xenografts: J7-sh-luc#1/vc (n = 4), J7-sh-luc#1/HMGA2 (n = 5), J7-shLOC344887#1/vc (n = 5), and J7-shLOC344887#1/HMGA2 (n = 5). Dotted lines indicate tumors in the lung tissues. The intensity of p-STAT3 and HMGA2 was assessed using IHC staining, and the quantitative results for each gene are presented in relative fold changes to vc, showing significantly elevated p-STAT3 when HMGA2 was overexpressed. The metastatic index (relative fold change) was determined for HMGA2-overexpressing versus vc with or without LOC344887 knockdown. Indicated scale bar is 50 μm. Statistical significances were obtained from one-way ANOVA followed by Tukey's post-hoc test. (G) J7 control cells (sh-luc#1) and LOC344887-depleted cells (shLOC344887#1) were transfected with HMGA2 reporter plasmids of varying promoter fragments (denoted by I to VII), containing -2000 bp to +1 bp HMGA2 promoter region relative to transcription start site. The HMGA2 luciferase reporter activities was measured and normalized to the control group (sh-luc#1), fold changes in reporter activity impacted by LOCK344887 knockdown were presented. Statistical significances were derived from one-way ANOVA followed by Tukey's post-hoc test. (H) Huh7 cells transfected with vector control (vc), LOC344887-v1 or LOC344887-v2 were immunoprecipitated with IgG or STAT3 antibody to detect binding signals at HMGA2 promoter region VI by PCR and gel electrophoresis (amplicon: F/R), with GAPDH used as a negative control. The fold enrichment from the ChIP assay (n = 3) was analyzed and presented. (I) The impacts of LOC344887 and STAT3 on HMGA2 mRNA expression were assessed in Huh7 cell lines with and without S3I-201 treatment (100 μM), showing HMGA2 as a downstream target of LOC344887-mediating STAT3 signaling. (J) The protein levels of p-STAT3 (Tyr705), STAT3 and HMGA2 were evaluated in vc- and Huh7 LOC344887-overexpressing cell lines, with or without S3I-201 treatments, showing significantly reduced protein levels of both p-STAT3 and HMGA2 when treated with S3I-201 (n = 3). *, P < 0.05; **, P < 0.01.

To substantiate the functional role of LOC344887/SHP-1/STAT3 signaling axis in HCC progression, microarray profiling data from LOC344887 stable knockdown cells alongside publicly available dataset GSE14520 24 were analyzed in aim to unravel potent genes regulated by this novel signaling axis in HCC. As shown in supplementary Figure 4A and 4B, significantly dysregulated gene expression in six potential molecules including HMGA2, TFPI, ARPP19, SEC14L2, FXYD1 and KBTBD11 were identified. The results showed that HMGA2, TFPI, and ARPP19 were highly expressed in HCC tissues, while expression of SEC14L2, FXYD1, and KBTBD1 was significantly lower in HCC as compared to normal tissues (Supplementary Figure 4C). Of note, higher expression of HMGA2 and TFPI was significantly correlated with poor prognosis, while elevated expression of SEC14L2 was associated with a better prognosis (Supplementary Figure 4D). QRT-PCR was performed to verify their expression levels and clinical significance in HCC. As expected, HMGA2 and TFPI were upregulated in HCC specimens (Figure 6A). However, a lower abundance of SEC14L2 led to barely detectable levels in HCC samples (Data not shown). Subsequently, the correlation between LOC344887, HMGA2, and TFPI in HCC was analyzed. The expression levels of LOC344887 were positively associated with HMGA2 but not TFPI (Supplementary Figure 4E). Moreover, higher expression of HMGA2 was correlated with poorer overall survival (Figure 6B). To consolidate HMGA2 as a target gene of this signaling axis, mRNA and protein expression of HMGA2 under the context of LOC344887 knockdown and overexpression were first examined. Our results demonstrated that LOC344887 depletion in J7 cells significantly reduced HMGA2 expression at both mRNA and protein levels, while upregulation of LOC344887-v1/-v2 significantly enhanced HMGA2 protein expression (Figure 6C). Further, SHP-1 knockdown led to significant increases in HMGA2 expression, which was conversely downregulated when SHP-1 was overexpressed (Supplementary Figure 5A and 5B). In addition, ectopic expression of the STAT3 mutant (Y705F) resulted in attenuated HMGA2 expression (Supplementary Figure 5C), suggesting that STAT3 phosphorylation at Tyr705 and SHP-1 both played a prominent role in HMGA2 regulation.

### LOC344887-mediated HCC pathogenesis is pertinently associated with transcriptional regulation of HMGA2 by STAT3

**Figure 7:**
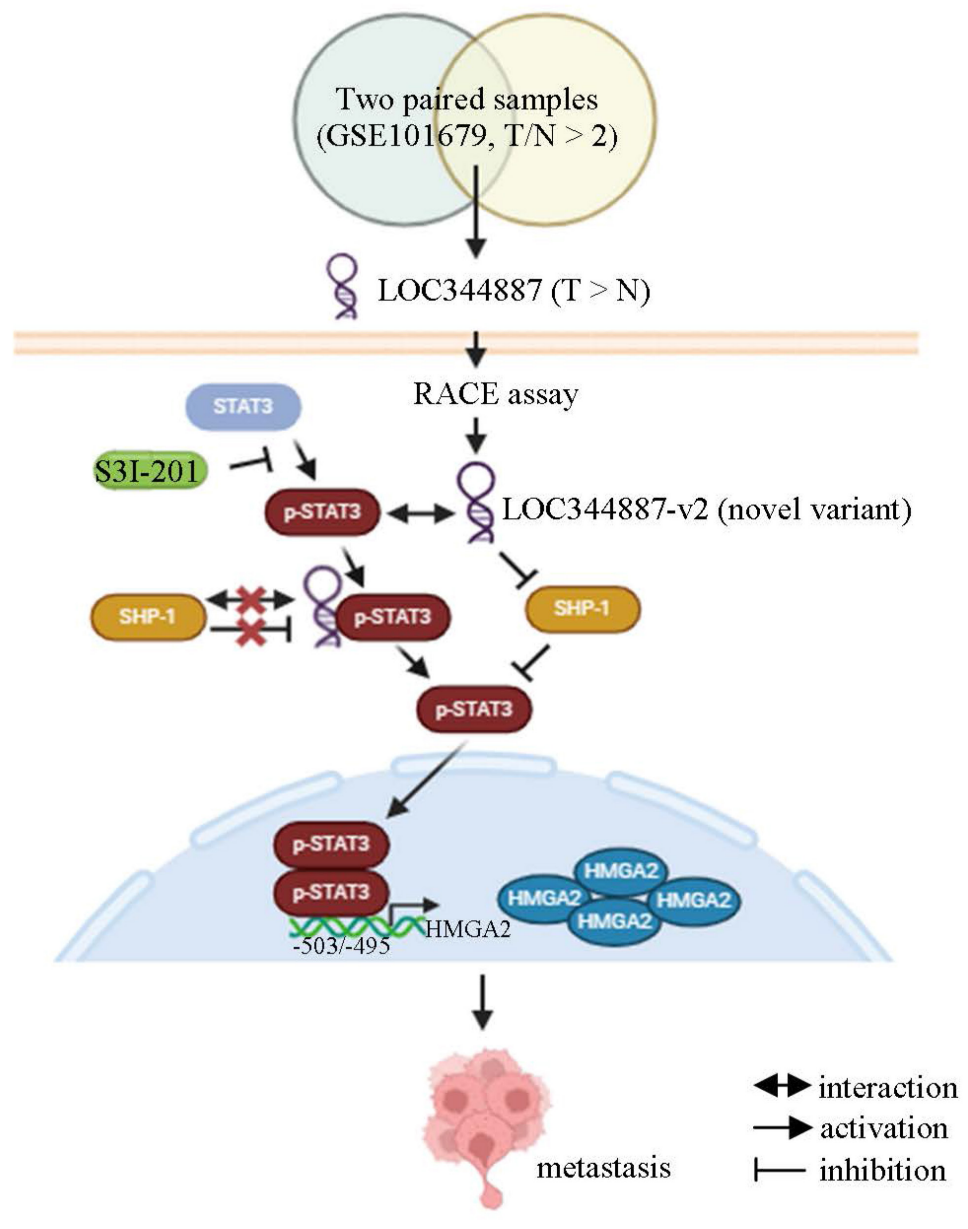
** Current model for LOC344887/SHP-1/STAT3/HMGA2 signaling cascade in regulating the metastatic potential in HCC.** Analysis of online datasets and qRT-PCR validation revealed LOC344887 as the prominent lncRNA highly expressed in HCC tissues. Elevated expression of LOC344887-v2 transcript manifested a more potent ability in activating STAT3 phosphorylation by repressing SHP1 expression and disrupting its interaction with STAT3, resulting in more robust STAT3 phosphorylation (Tyr705) than LOC344887-v1. The phosphorylated STAT3 (inhibitable by STAT3-specific inhibitor S3I-201) subsequently leads to upregulation of oncogene HMGA2, thereby promoting cell motility for HCC. Created with BioRender.com.

Overexpression of HMGA2 promoted cell migration in hepatoma cell lines (Figure 6D). To further address whether HMGA2 was a potential target involved in LOC344887-mediated cellular functions, *in vitro* and *in vivo* experiments were conducted in cell lines simultaneously depleted of LOC344887 and overexpressing HMGA2. The results showed that knockdown of LOC344887 repressed cell motility *in vitro* and *in vivo* (Figure 6E and Figure 6F). These phenotypes were partially reversed upon overexpression of HMGA2 (Figure 6E and Figure 6F). Additionally, our observations showed that mitigated STAT3 phosphorylation in lung tissues by LOC344887 knockdown could be significantly restored when HMGA2 was overexpressed in the xenografts (Figure 6F). These findings were supported by our HCC tissue array analysis, which demonstrated significantly elevated STAT3 phosphorylation and HMGA2 expression in tissues of HCC as compared to those of cirrhosis or normal (Supplementary Figure 6A). Consistently, correlation analysis on the tissue array data revealed significant associations between LOC344887 expression and protein levels of p-STAT3 (Tyr705) and HMGA2 (Supplementary Figure 6B). Moreover, since STAT3 has been a renowned transcription factor that modulates downstream gene expression influencing cellular growth, metastasis, metabolism and drug resistance 25, we next investigated whether STAT3 could directly bind to the promoter of HMGA2 and regulate its expression in HCC. Predicted STAT3 binding sites within the HMGA2 promoter region up to -2000 bp were identified, and seven luciferase reporter constructs (I to VII) including two mutants (V and VII) were designed to uncover STAT3 binding elements critical for LOC344887 modulation (Figure 6G). Results from the reporter activity assays demonstrated that LOC344887 knockdown resulted in a significant loss of report activity from construct II and IV, implicating -833/+1 as the promoter region that contained critical STAT3 binding elements for LOC344887 (Figure 6G). Further, the recovered reported activities from STAT3-2 mutant constructs V and VII in LOC344887-knockdown cells unravel -503/-495 as the critical STAT3 binding elements for LOC344887. Similar results were observed in LOC344887-depleted Mahlavu cell lines (Supplementary Figure 7A). Additionally, the transcriptional activity of HMGA2 was also enhanced by overexpression of LOC344887 transcripts compared to vc (Supplementary Figure 7B). Furthermore, the regulatory role of LOC344887/STAT3 in HMGA2 gene expression through STAT3-binding element within HMGA2 promoter was corroborated by chromatin immunoprecipitation (ChIP) assays, which manifested significantly enhanced *in vivo* binding of STAT3 to the HMGA2 promoter in the overexpression of LOC344887-v2 cells as compared to the control or LOC344887-v1 cells (Figure 6H). Notably, LOC344887-mediated HMGA2 induction at both mRNA (Figure 6I) and protein (Figure 6J) levels were significantly attenuated when STAT3 activity was attenuated by S3I-201 treatment. Together, present findings implicated a novel signaling axis orchestrated by LOC344887, with an emphasis on LOC344887-v2, which elicited more potent activation of STAT3 signaling, prevention of SHP-1 dephosphorylation and upregulation of HMGA2 in driving HCC migration and metastasis (Figure 7).

## Discussion

Our current study identifies the pseudogene-derived lncRNA LOC344887 as a significant prognostic factor in HCC. Consistent with previous studies that have highlighted the dysregulation of pseudogene-derived lncRNAs across various cancer types, including non-small cell lung and ovarian cancers 26, 27, our microarray profiling analysis conducted in this study showed 24 pseudogene-derived lncRNAs significantly upregulated in HCC, with LOC344887 being the most differentially expressed (Figure 1 and Table 1). These findings reveal that markedly upregulated LOC344887 in HCC tissues, as validated by qRT-PCR and ISH, was significantly correlated with poor OS ad RFS. This aligns with the growing body of literature suggesting that lncRNAs derived from pseudogenes can play pivotal roles in cancer progression, acting as oncogenes or tumor suppressors depending on the cellular context 28, 29. The identification of LOC344887 as a prognostic marker thus emphasizes the potential of pseudogene-derived lncRNAs in clinical settings, particularly in HCC, where early detection and prevention are critical for improving patient outcomes.

Functional assays conducted demonstrate that elevated LOC344887 expression greatly enhanced the migratory abilities of Huh7 and Hep3B cells. In contrast, the knockdown of LOC344887 in J7 and Mahlavu cells resulted in significantly reduced cellular migration, invasion, in vivo tumor growth, and lung metastasis (Figure 2). These results align with findings from other studies that have shown lncRNAs can facilitate cancer cell motility and metastasis through various mechanisms, including modulation of signaling pathways and interaction with key regulatory proteins 30, 31. The observed differences in migratory capacity between LOC344887 variants, with LOC344887-v2 exhibiting more pronounced effects, suggest that specific lncRNA isoforms may have distinct functional roles in cancer progression, which warrants further exploration.

Further, present findings in our study elucidate the underlying mechanisms by which LOC344887 is utilized to mediate its oncogenic effects through regulation of STAT3 phosphorylation at tyrosine 705 (Figure 3). The significant reduction in p-STAT3 levels upon LOC344887 knockdown indicates that LOC344887 is crucial for maintaining STAT3 activation in HCC. This finding is particularly relevant given the established role of the JAK/STAT pathway in HCC oncogenesis 32-34. The ability of LOC344887 to enhance STAT3 phosphorylation aligns with previous research indicating that lncRNAs can modulate signaling pathways by interacting with phospho-proteins to prevent their dephosphorylation 20, 21.

Moreover, the interaction of LOC344887 with p-STAT3, as demonstrated through RNA pull-down and RNA immunoprecipitation (RIP) assays (Figure 4), elicits the potential of lncRNAs to directly influence the activity of key signaling molecules. The stronger binding affinity of LOC344887-v2 to p-STAT3 compared to LOC344887-v1 suggests that variant-specific interactions may play a critical role in the regulation of STAT3 signaling, which is known to drive various aspects of tumor biology, including proliferation, survival, and metastasis. This variant-specific modulation of STAT3 signaling by LOC344887 is a novel finding that imposes significant implications for deeper understanding the functional diversity of lncRNAs in cancer.

Furthermore, our investigation into the role of SHP-1 in the regulation of STAT3 dephosphorylation uncovers that not only expression of SHP-1 could be regulated by LOC344887, but the stronger interaction of LOC344887-v2 with p-STAT3 than LOC344887-v1 prevented association and dephosphorylation of STAT3 by SHP-1 (Figure 5), suggesting the ability of LOC344887 in inhibiting SHP-1 activity to sustain STAT3 phosphorylation. This interplay between LOC344887, SHP-1, and STAT3 thus highlights the complexity of lncRNA-mediated regulation of signaling pathways, where lncRNAs can act as scaffolds to modulate the activity of phosphatases and kinases 20, 35, 36. Notably, the restoration of cell motility in LOC344887-depleted cells upon SHP-1 knockdown reinforces the notion that the LOC344887/SHP-1/STAT3 axis is crucial for HCC progression.

Finally, the identification of oncogene HGMA2 as a target of the LOC344887/SHP-1/STAT3 signaling axis provides further insights into the molecular mechanisms driving HCC (Figure 6, Supplementary Figures 6-7). The correlation between HMGA2 expression and poor prognosis in HCC patients aligns with previous studies that have implicated HMGA2 in promoting tumor growth and metastasis 37, 38. The oncogenic effects of LOC344887 were corroborated as the downstream effector role of HMGA2 was manifested by findings derived from HMGA2 reporter and ChIP assays, which demonstrated direct binding of STAT3 to the HMGA2 promoter region (-503/-495). Consistent with previous studies, the HMGA2 oncogenic pathway is shown to be enhanced by STAT3 signaling modulated Let-7a expression in breast cancer and glioblastoma cells 39-41. Recently, another study by Wang *et al*. demonstrates a novel association of HMGA2 with STAT3 promoter that induces STAT3 expression and subsequent macrophage recruitment 42, suggesting a potential positive feedback loop between STAT3 and HMGA2 during cancer progression. The ability of LOC344887 to regulate HMGA2 expression through STAT3 binding to its promoter thus accentuates the potential of targeting this axis for therapeutic intervention in HCC.

In conclusion, the findings of this study contribute to the growing body of evidence supporting the role of pseudogene-derived lncRNAs in cancer progression of HCC. The identification of LOC344887 as a prognostic factor and its involvement in regulating key signaling pathways underlines the potential of lncRNAs as biomarkers and therapeutic targets in HCC. Of note, the pertinent association of LOC344887-v2 expression with p-STAT3 (Tyr705) and HMGA2 inductions collectively provide a further explanation for the poorer OS and RFS attributed to higher LOC344887-v2/-v1 expression. Hence, future studies should focus on elucidating the precise mechanisms by which LOC344887 and other lncRNAs modulate cancer biology, as well as exploring their potential utility in clinical applications.

## Supplementary Material

Supplementary figures and tables.

## Figures and Tables

**Table 1:** The significantly up-regulated pseudogene-derived lncRNA in HCC

Gene Name	T/N ratio (average)
**LOC344887**	**15.337**
DUSP5P	9.466
FLJ43315	8.817
LOC100288637	8.435
ANXA2P3	6.244
SLC6A10P	5.868
ANXA2P1	5.174
LOC645166	4.145
AURKAPS1	3.776
FER1L4	3.592
LOC100132724	3.221
DPY19L1P1	3.217
RPSAP52	2.951
RPL23P8	2.806
ARGFXP2	2.693
psiTPTE22	2.598
MSL3P1	2.431
LOC728743	2.425
RPS2P32	2.385
FLJ43681	2.355
HSP90AB2P	2.261
LOC100129138	2.248
RPL23AP64	2.222
ANKRD20A8P	2.206

**Table 2:** Clinicopathological correlations of LOC344887 in HCC specimens.

Parameters	n = 158	LOC344887 Mean*^a^* ± SE	*p^b^*
Age (years)			
<65	84	29.02 ± 6.264	0.2665
≥65	74	42.06 ± 17.14	
Gender			
Male	117	50.14 ± 14.28	0.4347
Female	41	17.73 ± 3.875	
Cirrhosis			
No	98	43.52 ± 11.78	0.2174
Yes	60	19.00 ± 5.036	
AFP			
<400 ng/ml	97	34.24 ± 11.00	0.6871
≥400 ng/ml	61	33.18 ± 8.104	
Viral status			0.5354
NBNC	40	47.18 ± 31.19	
HBV	48	27.74 ± 6.925	
HCV	50	35.40 ± 10.20	
HBV&HCV	20	38.06 ± 32.39	
Tumor type			
Solitary	103	31.98 ± 9.023	0.0132
Multiple	55	40.80 ± 10.42	
Tumor size			
<5 cm	61	21.53 ± 4.971	0.1651
≥5 cm	97	51.25 ± 16.39	
Vascular invasion			
No	48	14.90 ± 4.318	0.0014
Yes	110	53.51 ± 14.21	
Pathological stage			
I	75	14.30 ± 4.467	0.0032
II	51	61.08 ± 20.61	
III	32	36.25 ± 10.23	
Grading			
1	8	0.942 ± 0.253	0.0856
2	105	34.82 ± 9.754	
3	45	34.37 ± 10.86	

a: Mean of LOC344887 expression (T/N ratio) b: Mann-Whitney U test (for two groups) or Kruskal Wallis test (for > two groups)

## Abbreviations

MiRNA: microRNA
lncRNA: long non-coding RNA
HCC: hepatocellular carcinoma
PCNAP1: proliferating cell nuclear antigen pseudogene 1
LPAL2: lipoprotein(a) like 2
MMP9: matrix metalloproteinase 9
HULC: highly upregulated in liver cancer
STAT3: signal transducer and activator of transcription 3
SHP-1: src homology region 2 domain-containing phosphatase
NMRAL2P: nmrA like redox sensor 2, pseudogene
NRF2: nuclear factor erythroid 2-related factor 2
HMGA2: high mobility group AT-hook 2
qRT-PCR: quantitative real-time polymerase chain reaction
RACE: rapid amplification of cDNA ends
SCID: severe combined immunodeficient
RIP: RNA immunoprecipitation
TFPI: tissue factor pathway inhibitor
ARPP19: CAMP-regulated phosphoprotein 19
SEC14L2: SEC14 like lipid binding 2
FXYD1: FXYD domain containing ion transport regulator 1
KBTBD11: kelch repeat and BTB domain containing 11
ChIP: Chromatin immunoprecipitation
