# Supplementary material for: The novel role of LOC344887 in the enhancement of hepatocellular carcinoma progression via modulation of SHP1-regulated STAT3/HMGA2 signaling axis
Source: Int J Biol Sci. 2024 Dec 2;20(15):6281–96. doi: 10.7150/ijbs.99683 (PMC11628343; doi:10.7150/ijbs.99683)
Supplement: Supplementary file 1 — Supplementary figures and tables. [file ijbsv20p6281s1.pdf]

The novel role of LOC344887 in the enhancement of hepatocellular carcinoma progression via modulation of SHP1-regulated STAT3/HMGA2 signaling axis

Supplemental information

Supplementary materials and methods

Cell culture and reagents

HepG2 (RRID:CVCL_0027), Huh7 (RRID: CVCL_0336), Hep3B (RRID:CVCL_0326), J7 (RRID:CVCL_4Z69)(gift from Dr. C S Yang, National Taiwan University, Taiwan)[1], Mahlavu (RRID:CVCL_0405), and SK-Hep1 (RRID:CVCL_0525) were cultured in Dulbecco's Modified Eagle's Medium (DMEM; Gibco, New York, USA) supplemented with 10% fetal bovine serum (FBS; Gibco), 100 µg/mL streptomycin sulfate, and 100 IU/mL penicillin G. Cells were maintained in an incubator at 37℃ with a humidified atmosphere and 5% CO2. Prior to conducting in vitro assays, the cells were subjected to examination for mycoplasma contamination using the TOOLS mycoplasma detection kit (BIOTOOLS, New Taipei City, Taiwan, TTB-GBC8) to ensure their integrity.

RNA extraction, reverse transcription polymerase chain reaction (RT-PCR) and qRT-PCR

To ascertain the transcript of the target gene, total RNA was isolated using TRIzol reagent (Life Technologies Inc., Carlsbad, CA, USA). Subsequently, the RNA was converted into cDNA through reverse transcriptase (Life Technologies). For the RT-PCR analysis, a 20 μL reaction was conducted with forward and reverse primers and 1X DNA polymerase. The RT-PCR conditions included an initial denaturation at 95℃ for 5 min, followed by 30 cycles of 95℃ for 30 sec, 55℃ for 30 sec, and 72℃ for 1 min. For the qRT-PCR analysis, a 15 μL reaction was performed with forward and reverse primers and 1X SYBR green master mix (Applied Biosystems, Carlsbad, CA, USA). The standard qRT-PCR conditions were an initial denaturation at 95℃ for 10 min, followed by 40 cycles of 95℃ for 15 sec, and 60℃ for 1 min. The primers utilized in this study are listed in Supplementary Table 1.

5′ and 3′ rapid amplification of cDNA ends (RACE) assays

RACE experiments (Roche Diagnostics, Mannheim, Germany, Catalog No. 03343621001) followed the manufacturer's protocol. Briefly, the amplification of the LOC344887 signal was performed through nested PCR using two sets of primers, as listed in Supplementary Table 1. The PCR product was visualized by electrophoresis and ligated into the pGEM-T easy vector for DNA sequencing. The sequences derived from the sequencing results were aligned using the NCBI genome browser.

Immunoblot analysis

The immunoblot analysis protocol was executed following previously described procedures [2]. The antibodies employed in this study are detailed in Supplementary Table 2. Signal detection was accomplished using X-ray films for chemiluminescence detection. Signal intensities were quantified using Image Gauge software. The expression levels of the target genes were normalized to GAPDH.

***In situ* hybridization**

The detection of the LOC344887 signal was performed using the RNAScope^{TM} Detection Reagent Kit RED with a custom LOC344887 probe (Advanced Cell Diagnostics, Hayward, CA, USA, #323970-USM). The LOC344887 signal was visualized as a brown signal. The stained tissue array was scanned and observed using NDP.view2 viewer. The intensity of LOC344887 in the tissue array was quantified using Image J software.

Immunohistochemical (IHC) staining

The immunohistochemical staining protocol has been previously described [3]. Briefly, the primary antibodies used for IHC staining and their dilutions are listed in Supplementary Table 2. Detection was performed using the EnVision kit (DAKO,

Carpinteria, CA, USA), with positive signals appearing as brown. The intensity of p-STAT3 (Tyr705) and HMGA2 in tumor cells was categorized as absent (0), weak (1+), moderate (2+), or strong (3+). Additionally, the percentage of positive cells was classified as absent (0), focal (≤10%), regional (11%-50%), or diffuse (>50%). The histoscore was calculated by multiplying the percentage of antibody-positive cells by the intensity.

Gene expression and pathway analysis

Total RNA from LOC344887 knockdown J7 cell lines was isolated using TRIzol reagent and analyzed for gene expression profiling with the Agilent SurePrint G3 Gene Expression Microarray (Agilent Technologies, Santa Clara, CA, USA). Signal extraction was carried out using Feature Extraction software (version 10.7.3.1). Differentially expressed genes regulated by LOC344887 were identified using their official gene IDs and further subjected to KEGG pathway analysis using the clusterProfiler (4.8.2) package.

Cell proliferation

LOC344887 stable cells (5×10^4) were seeded in 6 cm plates. After 24h, 48h, and 72h of seeding, the cells stained with 0.4% trypan blue were determined using a cell

counter. The cell growth rate was normalized to day one and presented as a fold change.

Cell migration and invasion assay

The Transwell assay (Becton-Dickinson, Franklin Lakes, NJ, USA, #3422) assessed cell migratory and invasive capabilities. Cells (8×10^2 cells/μl) suspended in serum-free medium were seeded in the upper chamber, which was either non-matrigel-coated (for migration assay) or matrigel-coated (for invasion assay). In contrast, the lower chamber contained DMEM with 20% FBS. After 18 hours of incubation, migratory or invasive cells were fixed and stained using crystal violet. Image J software quantified the cells traversing from the upper to the lower chamber.

Establishment of target gene stable cell lines

Full-length sequences of LOC344887 (v1 and v2), HMGA2, STAT3 (wild-type), and SHP-1 were amplified by PCR and cloned into pcDNA3 expression plasmid (Invitrogen, Carlsbad, CA, USA). The dominant-negative STAT3 (Y705F) pcDNA3 plasmid (Plasmid #74434) was purchased from Addgene. The pool stable overexpression of those genes in hepatoma cell lines was incubated with DMEM with 10% FBS and G418 (800 μg/ml). We also performed the transient transfection assays

(as described in the corresponding figure legend). The shRNAs targeted to the LOC344887 (shLOC344887#1 and shLOC344887#2) were synthesized and cloned into pLKO.1 vector based on the guidelines of the TRC shRNA design algorithm (RNAi core). In addition, the HMGA2 and SHP-1 shRNAs were purchased from the National RNA Interference Core Facility (Institute of Molecular Biology, Academia Sinica, Taiwan). The lentivirus-targeted individual genes were prepared as described previously [4]. The pool stable knockdown specific genes were incubated with DMEM with 10% FBS and puromycin (0.5 μg/ml). The information on each shRNA is listed in Supplementary Table 1.

Animal models

In tumor formation assay, Mahlavu-sh-luc#1 (1×10^6 cells, n = 4), Mahlavu-shLOC344887#1 (1×10^6 cells, n = 3), J7-sh-luc#1 (2×10^6 cells, n = 5) and J7-shLOC344887#1 (2×10^6 cells, n = 5) were injected into the nude mice, respectively. Tumor volumes were analyzed by the formula (W2xL)/2 (W, most minor diameter, and L, longest diameter). In the metastatic assay, severe combined immunodeficient (SCID) mice were used to investigate the metastatic effect of LOC344887-depleted J7 cells. J7-sh-luc#1 and J7-shLOC344887#1 (2×10^6, n = 4) cells were intravenously injected into the SCID mice. Furthermore, SCID mice were

used to uncover the metastatic effect of the LOC344887/HMGA2 axis (rescue experiment). J7-sh-luc#1/vc (n = 4), J7-shLOC344887#1/vc (n = 5), J7-sh-luc#1/HMGA2 (n = 5), and J7-shLOC344887#1/HMGA2 (n = 5) cells (2 × 10^6) were intravenously injected into the SCID mice. After H&E staining, the metastatic tumor (relative fold) in the lungs was determined. All assays were performed according to the United States National Institutes of Health guidelines and Chang Gang Institutional Animal Care and Use Committee Guide for the Care and Use of Laboratory Animals (IACUC: CGU109-135).

Human JAK/STAT Pathway Phosphorylation Array

The human JAK/STAT pathway phosphorylation array kit (Catalog #AAH-JAKSTAT-1-4) was used following the manufacturer's protocol (RayBiotech Life, Inc., Peachtree Corners, GA, USA). Briefly, 150 μg of protein extracted from control and LOC344887 knockdown cell lines was incubated with a blocking membrane overnight at 4℃. The membranes were then washed with wash buffers I and II. Afterward, the detection antibody cocktail was incubated with the membranes overnight at 4℃. An HRP-conjugated antibody was applied for 2 hours at room temperature. Chemiluminescence was used to detect the signals visualized on X-ray film.

Reporter assay

The vector NTI software (Thermo Fisher Scientific) was employed to predict six potential STAT3 binding sites (-1500/-1492, -912/-904, -866/-858, -503/-495, -440/-432, and -414/-406) within the HMGA2 promoter region. Fragments of the HMGA2 promoter (fragments I, II, III, IV, and VI) and a deletion of the putative STAT3 binding site in the HMGA2 promoter (fragments V and VII) were amplified using PCR and then cloned into the PGL3-TK vector. Hepatoma cells (3×10^4) were seeded in a 24-well plate and co-transfected with pcDNA3, pcDNA3-LOC344887-v1, pcDNA3-LOC344887-v2, HMGA2 reporter plasmids, and the β-galactosidase plasmid (Clontec Laboratories Mountain View, CA, USA) using TurboFectTM (Thermo Fisher Scientific). Luciferase activity was normalized to the control vector. In addition, the STAT3 reporter activity was determined using STAT3 Reporter Assay Kit (BPS Bioscience, San Diego, CA, USA, #79730).

Chromatin immunoprecipitation (ChIP) assay

This assay was performed on the HMGA2 promoter region following a previously described protocol [2]. Briefly, hepatoma cells overexpressing vector control, LOC344887-v1, and LOC344887-v2 were harvested and cross-linked with 1% formaldehyde. The reaction was terminated by adding 0.125M Glycine. After two

washes with ice-cold PBS, cells were lysed using RIPA buffer (25 mM Tris-HCl, pH 7.5, 150 mM NaCl, 1% Triton X-100, 1% Na deoxycholate, 0.1% SDS, and 2 mM EDTA, pH 8.0) containing protease inhibitors (Merck Millipore, #539134). Samples were sonicated with the Misonix Sonicator 3000 Homogenizer (Mandel Scientific Company Inc., Guelph, ON, Canada). To minimize non-specific binding, samples were incubated with magnetic protein A/G (GE Healthcare Life Sciences) for 1 hour at 4℃. Subsequently, the products were incubated overnight at 4℃ with STAT3 (ABclonal, A19566) and IgG (Cell signaling, #2729) antibodies. The complexes were precipitated by adding magnetic protein A/G for 1 hour at 4℃. After precipitation, samples were washed with wash buffer. DNA products were extracted and eluted using the QIAquick PCR purification kit (QIAGEN Inc., Valencia, CA, USA, Cat. No. 8106). The STAT3 binding fragments were identified using PCR, and the primer details are listed in Supplementary Table 1.

RNA pull-down assay

The sense and anti-sense strands of biotin-labeled LOC344887 were generated using the T7/SP6 transcription kit (Roche, Cat. No. 11685597910). The RNA lengths of LOC344887-v1 and LOC344887-v2 generated by in vitro transcription were verified using denaturing agarose gel electrophoresis with RNA Millennium™ markers (ABI,

#AM7150). The synthesized biotin-labeled RNA (3 μg) was incubated with total proteins (1000 μg). After a 1-hour incubation at room temperature, the complex was treated with ice-cold NT2 wash buffer and combined with 2x Laemmli loading buffer. The samples were then subjected to SDS-PAGE. The signals of p-STAT3 (Tyr705) and GAPDH were detected using X-ray films for chemiluminescence detection.

RNA immunoprecipitation (RIP) assay

The RIP assay was conducted following a previously described protocol [5]. Briefly, cells were washed with ice-cold PBS and lysed with ice-cold polysomal lysis buffer (100 mM KCl, 5 mM MgCl, 10 mM HEPES (pH 7.0), 0.5% NP-40, 1 mM DTT, RNase inhibitor (Invitrogen, Cat. No. 10777019), and protease inhibitors (Merck Millipore, #539134)). Samples were collected by centrifugation and pre-cleared with magnetic protein-A/G beads (GE Healthcare Life Sciences). Immunoprecipitation (IP) was carried out by adding STAT3 (ABclonal, A19566) and p-STAT3 Y705 antibody (ABclonal, AP0705) at 4℃ overnight. Each sample was incubated with magnetic protein A/G beads for 1 hour at 4℃. Subsequently, complexes were washed with the lysis buffer. To prevent DNA contamination, DNase I (Roche, Cat. No. 04716728001) was incubated for 15 minutes at 37℃ after washing. Total RNA was then extracted using Trizol reagent.

In vitro phosphatase assay

The *in vitro* phosphatase assay followed a previously described protocol [6, 7]. In brief, hepatoma cells were transfected with 3×FLAG-STAT3 plasmid for 72 h. After transfection, total cell lysates were extracted and incubated with anti-FLAG M2 magnetic beads (Sigma) for 4 h. Then, the aliquoted sample was mixed with recombinant human SHP-1 (R&D systems), LOC344887-v1, and v2 transcript. The p-STAT3 (Tyr705) and total STAT3 expressions were determined via immunoblot analysis.

Duolink proximity ligation assay

In situ proximity ligation assays (PLAs) were performed on fixed cells according to the manufacturer's protocol (DUO92008, Sigma-Aldrich). Coverslips were initially blocked for 45 minutes at 37℃ with a blocking solution and then incubated with a 1:200 dilution of primary antibodies. SHP-1 antibody (Cell Signaling Technology, #26516) alone was served as the negative control, while the interaction between SHP-1 and p-STAT3 (Tyr705) was evaluated using both SHP-1 and p-STAT3 (Merck, 05485) antibodies at 4℃ for overnight. Subsequently, the coverslips were washed three times for 10 minutes each with PBS containing 0.1% Tween 20, followed by incubation with secondary antibodies (PLA probes, Sigma-Aldrich) at 37 ℃ for 2

hours. After additional washing steps with buffer B, DNA ligase was added and incubated at 37 ℃ for 30 minutes. A subsequent amplification step involved incubation with DNA polymerase (DUO82030, Sigma-Aldrich) at 37 ℃ for 2 hours. Finally, the coverslips were rinsed with buffer A and washed with 0.1× buffer B before being mounted with a mounting solution containing DAPI. The close proximity of these proteins, defined as less than 40 nm, is visualized by discrete red fluorescent puncta under fluorescence microscopy.

Supplementary figure legends

Supplementary Figure 1. Full-length sequences of the two LOC344887 variants in HCC. Upper panel: A schematic representation of LOC344887 variants in the study, with the red line indicating the unique sequences for the v1 and v2 transcripts of LOC344887. Lower panel: The full nucleotide sequences of LOC344887-v1 and -v2 transcripts, identified by 5'RACE and 3'RACE, are shown. The distinctive sequences between the two variants are marked in red.

Supplementary Figure 2. Identification of LOC344887-mediated pathway. (A) A schematic workflow is illustrated for microarray profiling analysis using control (sh-luc#1) and LOC344887-depleted (shLOC344887#1) J7 stable cell lines. (B)

Differentially expressed genes from LOC344887-silenced (shLOC344887#1) stable cells as compared to control cells (sh-luc#1) were analyzed using KEGG pathway analysis, revealing the top enriched pathways dysregulated under LOC344887 knockdown. (C) A table showing enrichment scores for differentially regulated signaling pathways from LOC344887-depleted cells versus control cells.

Supplementary Figure 3. Knockdown and overexpression of SHP-1 reciprocally regulate cell motility of J7 cells.

The impacts of SHP-1 (A) knockdown or (B) overexpression on cell motility of J7 cells were evaluated by transducing J7 cells with two SHP-1-specific shRNA, which resulted in significantly increased numbers of migratory cells. Migratory cells stained with crystal violet were subjected to quantification using ImageJ software, and statistical significance was determined by one-way ANOVA followed by Tukey's post-hoc test. The scale bar indicates 100 μm. **, $P < 0.01$.

Supplementary Figure 4. Data analysis on LOC344887 knockdown microarray data and GSE14520 identifies six potently dysregulated genes.

(A) RNA was extracted from control (sh-luc#1) and LOC344887-depleted (shLOC344887#1) cells and analyzed via microarray profiling. A Venn diagram

analysis revealed six differentially expressed genes in LOC344887-depleted J7 cells and HCC dataset GSE14520 (intersection region), including high mobility group AT-hook 2 (HMGA2), Tissue Factor Pathway Inhibitor (TFPI), CAMP-Regulated Phosphoprotein 19 (ARPP19), SEC14 Like Lipid Binding 2 (SEC14L2), FXYD Domain Containing Ion Transport Regulator 1 (FXYD1) and Kelch Repeat and BTB Domain Containing 11 (KBTBD11). The selection criteria for target genes were: (1) LOC344887#1/sh-luc#1 ratio > 2, with $P < 0.05$ and $T/N < 0.83$, and (2) LOC344887#1/sh-luc#1 ratio < 0.5, with $P < 0.05$ and $T/N > 1.2$. (B) Relative fold-change (shLOC344887#1/sh-luc#1) in the expression of the six potential target genes regulated by LOC344887 are shown. (C) The expression intensity of the six target genes in normal tissues (N) and HCC tissues (T) was examined. Statistical significances were derived from the Mann-Whitney test. (D) Overall survival and recurrence-free survival in relation to the expression of the six candidates were analyzed using the publicly available GSE14520 dataset with Kaplan-Meier survival analysis and log-rank tests, showing HMGA2 and TFPI as only two candidates correlated with worse OS and RFS. Median expression values of these genes were used as cutoffs. *, $P < 0.05$; **, $P < 0.01$. (E) The expression correlations between LOC344887, HMGA2, and TFPI in HCC tissues were determined using Spearman's rank correlation coefficient.

Supplementary Figure 5. HMGA2 expression can be regulated by phosphatase SHP-1 and STAT3 phosphorylation.

Impacts of SHP-1 on HMGA2 expression were investigated by (A) knocking down or (B) overexpressing SHP-1 and assessing for changes in HMGA2 protein levels. (C) A dominant negative mutant of STAT3 (Y705F) was utilized to assess the role of STAT3 in mediating HMGA2 expression.

Supplementary Figure 6. Clinicopathological correlations of LOC344887 to p-STAT3 (Tyr705) and HMGA2 using HCC tissue array.

(A) HCC tissue arrays that contain normal, cirrhosis, and HCC were utilized to examine their correlation to p-STAT3 (Tyr705) and HMGA2 expression using immunohistochemistry (IHC). The brown signals indicated the expression of p-STAT3 (Tyr705) and HMGA2, showing significantly elevated expression of p-STAT3 and HMGA2 in HCC when compared to normal or cirrhosis tissues—scale bar: 100 μm. Quantitative results and statistical significance were determined using one-way ANOVA followed by Tukey's post-hoc test. *, $P < 0.05$; **, $P < 0.01$. (B) The correlation between LOC344887 (RNA level), p-STAT3 (Tyr705) (protein level), and HMGA2 (protein level) in HCC was evaluated using Spearman's rank correlation coefficient, showing significant correlations among LOC344887, p-STAT3 (Tyr705)

and HMGA2.

Supplementary Figure 7. HMGA2 promoter contains putative STAT3 binding element responsible for LOC344887 in HCC.

(A) Mutant construct (VII) and wild-type construct (VI) containing putative STAT3-2 binding site were transfected into Mahlavu control cells (sh-luc#1) or LOC344887-depleted cells (shLOC344887#1) to demonstrate the importance of STAT3-2 in LOC344887-mediated HMGA2 promoter activity. (B) The same reporter constructs utilized in (A) were transfected into control (vc), LOC344887-v1 or LOC344887-v2-overexpressing Huh7 cells, showing significantly enhanced HMGA2 promoter activities when either LOC344887 variant was overexpressed. Luciferase reporter activities measured were normalized to vc and presented in fold changes, as statistical significance was obtained from one-way ANOVA followed by Tukey's post-hoc test. *, $P < 0.05$; **, $P < 0.01$.

References

1. Hosono S, Lee CS, Chou MJ, Yang CS, Shih CH. Molecular analysis of the p53 alleles in primary hepatocellular carcinomas and cell lines. Oncogene. 1991; 6: 237-43.

2. Lin YH, Wu MH, Liu YC, Lyu PC, Yeh CT, Lin KH. LINC01348 suppresses hepatocellular carcinoma metastasis through inhibition of SF3B3-mediated EZH2 pre-mRNA splicing. Oncogene. 2021; 40: 4675-85.

3. Liao CJ, Wu TI, Huang YH, Chang TC, Wang CS, Tsai MM, et al. Glucose-regulated protein 58 modulates cell invasiveness and serves as a prognostic marker for cervical cancer. Cancer Sci. 2011; 102: 2255-63.

4. Chan YL, Liao CL, Lin YL. Human Kinase/Phosphatase-Wide RNAi Screening Identified Checkpoint Kinase 2 as a Cellular Factor Facilitating Japanese Encephalitis Virus Infection. Front Cell Infect Microbiol. 2018; 8: 142.

5. Hsieh CL, Liu H, Huang Y, Kang L, Chen HW, Chen YT, et al. ADAR1 deaminase contributes to scheduled skeletal myogenesis progression via stage-specific functions. Cell Death Differ. 2014; 21: 707-19.

6. Kim BR, Ha J, Kang E, Cho S. Regulation of signal transducer and activator of transcription 3 activation by dual-specificity phosphatase 3. BMB Rep. 2020; 53: 335-40.

7. Liu C, Shen A, Song J, Cheng L, Zhang M, Wang Y, et al. LncRNA-CCAT5-mediated crosstalk between Wnt/beta-Catenin and STAT3 signaling suggests novel therapeutic approaches for metastatic gastric cancer with high Wnt activity. Cancer Commun (Lond). 2024; 44: 76-100.

Supplementary Figures

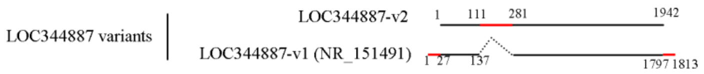

LOC344887-v1

ATAATTCTCACCAGAGAAGAAACACAGCATTTCGGCTGGTGTCGCTGCTCCAGGG
AAAGTTCTGTTACTCCACTGACTCTCTCTTTTCCTGATAACATGGCCAGCAAGAAA
GTAATTACAGTGTTTGGAGCAACAGGGGAAGCTGGTGGCAGACTCCGCCAAGCA
CCTGGGTCTGAAGCACGTGGTGTACAGCGGCCTGGAGAACGTCAAGCGACTGAC
GGATGGCAAGCTGGAGGTGCCGCACTTTGACAGCAAGGGCGAGGTGGAGGAGTA
CTTCTGGTCCATTGGCATCCCCATGACCAGTGTCCGCGTGGCGGCCTACTTTGAA
AACTTTCTCGCGGCGTGGCGGCCCGTGAAAGCCTCTGATGGAGATTACTACACCT
TGGCTGTACCGATGGGAGATGTACCAATGGATGGTATCTCTGTTGCTGATATTGG
AGCAGCCGTCTCTAGCATTTTTAATTCTCCAGAGGAATTTTTAGGCAAGGCCGTG
GGGCTCAGTGCAGAAGCACTAACAATACAGCAATATGCTGATGTTTTGTCCAAGG
TTTTGGGGAAAGAAGTCCGAGATGCAAAGATTACCCCGGAAGCTTTCGAGAAGC
TGGGATTCCCTGCAGCAAAGGAAATAGCCAATATGTGTCGTTTCTATGAAATGAA
GCCAGACCGAGATGTCAATCTCACCCACCAACTAAATCCCAAAGTCAAAAGCTTC
AGCCAGTTTATCTCAGAGAACCAGGGAGCCTTCAAGGGCATGTAGAAAATCAGC
TGTTCAGATAGGCCTCTGCACCACACAGCCTCTTTCCTCTCTGATCCTTTTCCTCTT
TACGGCACAACATTCATGTTGACAGAACATGCTGGAATGCAATTGTTTGCAACAC
CGAAGGATTTCCTGCGGTCGCCTCTTCAGTAGGAAGCACTGCATTGGTGATAGAA
CACGGTAATTTGATTCACATTTAACTTGCTAGTTAGTGATAAGGGTGGTACACCT
GTTTGGTAAAATGAGAAGCCTCGGAACTTGGAGCTTCTCTCCTACCACTAATGGG
AGGGCAGATTATACTGGGATTTCTCCTGGGTGAGTAATTTCAAGCCCTAATGCTG
AAATTCCCCTAGGCAGCTCCAGTTTTCTCAACTGCATTGCAAAATTCCCAGTGAA
CTTTTAAGTACTTTTAACTTAAAAAAATGAACATCTTTGTAGAGAATTTTCTGGGG
AACATGGTGTTCAATGAACAAGCACAAGCATTGGAAATGCTAAAATTCAGTTTTG
CCTCAAGATTGGAAGTTTATTTTCTGACTCATTCATGAAGTCATCTATTGAGCCAC
CATTCAATTATTCATCTATTAATTCCTTGATCCTTCATTTATCCATTCTGCAAACTT
TTCTTGAGCACCAGCACGGGTGGCCATTTGTGGACTTCTCTTCATTCCTATGTGTT
TTCTTATCAAAGTGATCCACTCTCGAAAGGCTCCTTTCCAGTCTGTGGTTGGGTTC
AAGTCATGCCAGGGCCAGGGGGCCCATCTCCTCGTTTAGCTCTAGGCAAAATCCA
GGGGATCTGCAGTGGGGAGCGGGGGCAGGAAGCTGGAGGGAAGGCCTGTGAAG
GGTAGGGATGTGGAAAGACAAGGTGACAGAAGGACCCAATAGGACCTTTCTATA
TCTCTGGCTTAGCATTTTCTACATCATATTGTAATCGTCTTATTTGCTAGTTTTCTT
CCTTACTGTGAGTGACTAACAGTCATCTTTATCCCAGTGCCTGGTACATAATAAGT
GATCAATAAATGTTGATTGACTAAATGAGTAAAAAAAAAAAAAAAAAAA

LOC344887-v2

GCATTTCGGCTGGTGTCGCTGCTCCAGGGAAAGTTCTGTTACTCCACTGACTCTCT
CTTTTCCTGATAACATGGCCAGCAAGAAAGTAATTACAGTGTTTGGAGCAACAGG
AGCTCAAGGTGGCTCTGTGGCCAGGGCAATTTTGGAGAGCAAAAAATTTGCAGT
GAGAGCAGTGACCAGGGATGTGACTTGACCAAATGCCCTGGAGCTCCAGCGCCT
TGGAGCTGAGGTGGTCAAAGGTGACCTGAATGATAAAGCATCGGTGGACAGTGC
CTTAAAAGGGGAAGCTGGTGGCAGACTCCGCCAAGCACCTGGGTCTGAAGCACG
TGGTGTACAGCGGCCTGGAGAACGTCAAGCGACTGACGGATGGCAAGCTGGAGG
TGCCGCACTTTGACAGCAAGGGCGAGGTGGAGGAGTACTTCTGGTCCATTGGCAT
CCCCATGACCAGTGTCCGCGTGGCGGCCTACTTTGAAAACTTTCTCGCGGCGTGG
CGGCCCGTGAAAGCCTCTGATGGAGATTACTACACCTTGGCTGTACCGATGGGAG
ATGTACCAATGGATGGTATCTCTGTTGCTGATATTGGAGCAGCCGTCTCTAGCATT
TTTAATTCTCCAGAGGAATTTTTAGGCAAGGCCGTGGGGCTCAGTGCAGAAGCAC
TAACAATACAGCAATATGCTGATGTTTTGTCCAAGGTTTTGGGGAAAGAAGTCCG
AGATGCAAAGATTACCCCGGAAGCTTTCGAGAAGCTGGGATTCCCTGCAGCAAA
GGAAATAGCCAATATGTGTCGTTTCTATGAAATGAAGCCAGACCGAGATGTCAAT
CTCACCCACCAACTAAATCCCAAAGTCAAAAGCTTCAGCCAGTTTATCTCAGAGA
ACCAGGGAGCCTTCAAGGGCATGTAGAAAATCAGCTGTTCAGATAGGCCTCTGC
ACCACACAGCCTCTTTCCTCTCTGATCCTTTTCCTCTTTACGGCACAACATTCATG
TTGACAGAACATGCTGGAATGCAATTGTTTGCAACACCGAAGGATTTCCTGCGGT
CGCCTCTTCAGTAGGAAGCACTGCATTGGTGATAGAACACGGTAATTTGATTCAC
ATTTAACTTGCTAGTTAGTGATAAGGGTGGTACACCTGTTTGGTAAAATGAGAAG
CCTCGGAACTTGGAGCTTCTCTCCTACCACTAATGGGAGGGCAGATTATACTGGG
ATTTCTCCTGGGTGAGTAATTTCAAGCCCTAATGCTGAAATTCCCCTAGGCAGCTC
CAGTTTTCTCAACTGCATTGCAAAATTCCCAGTGAACTTTTAAGTACTTTTAACTT
AAAAAAATGAACATCTTTGTAGAGAATTTTCTGGGGAACATGGTGTTCAATGAAC
AAGCACAAGCATTGGAAATGCTAAAATTCAGTTTTGCCTCAAGATTGGAAGTTTA
TTTTCTGACTCATTCATGAAGTCATCTATTGAGCCACCATTCAATTATTCATCTAT
TAATTCCTTGATCCTTCATTTATCCATTCTGCAAACTTTTCTTGAGCACCAGCACG
GGTGGCCATTTGTGGACTTCTCTTCATTCCTATGTGTTTTCTTATCAAAGTGATCC
ACTCTCGAAAGGCTCCTTTCCAGTCTGTGGTTGGGTTCAAGTCATGCCAGGGCCA
GGGGGCCCATCTCCTCGTTTAGCTCTAGGCAAAATCCAGGGGATCTGCAGTGGGG
AGCGGGGGCAGGAAGCTGGAGGGAAGGCCTGTGAAGGGTAGGGATGTGGAAAG
ACAAGGTGACAGAAGGACCCAATAGGACCTTTCTATATCTCTGGCTTAGCATTTT
CTACATCATATTGTAATCGTCTTATTTGCTAGTTTTCTTCCTTACTGTGAGTGACTA
ACAGTCATCTTTATCCCAGTGCCTGGTACATAATAAGTGATCAATAAATGTTGAT
TGACTAAATGAGTAAA

Supplementary Figure 1

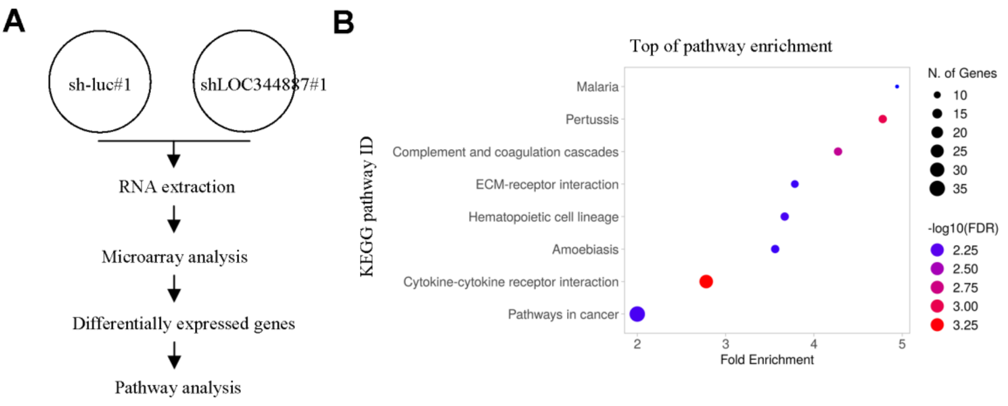

C

The differential signaling in LOC344887-depleted J7 and control cell

Signaling	Fold enrichment	Enrichment FDR
KEGG_Malaria	4.94136	0.00671
KEGG_Pertussis	4.77882	0.001
KEGG_Complement and coagulation cascades	4.27282	0.00215
KEGG_ECM-receptor interaction	3.78323	0.00622
KEGG_Hematopoietic cell lineage	3.66859	0.00596
KEGG_Amoebiasis	3.56069	0.00622
KEGG_Cytokine-cytokine receptor interaction	2.77951	0.00054
KEGG_Pathways in cancer	1.99868	0.00596

Abbreviations: KEGG, Kyoto encyclopedia of genes and genomes.

Supplementary Figure 2

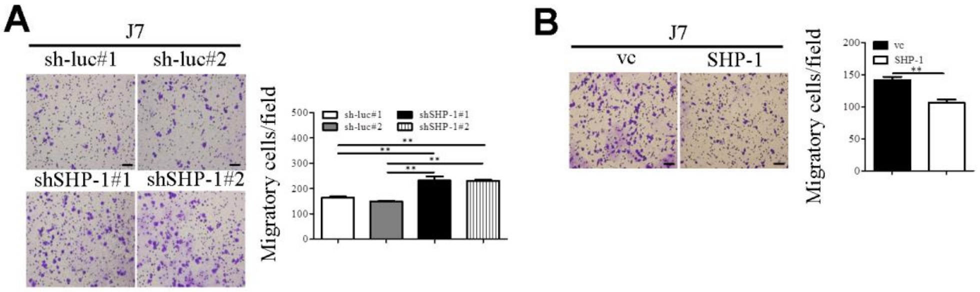

Supplementary Figure 3

A

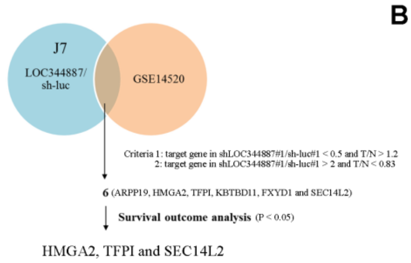

B

Gene symbol	Relative fold-change (shLOC344887#1/sh-luc#1)
HMGA2	0.44
TFPI	0.33
ARPP19	0.48
SEC14L2	2.09
FXYD1	2.006
KBTBD11	2.009

C

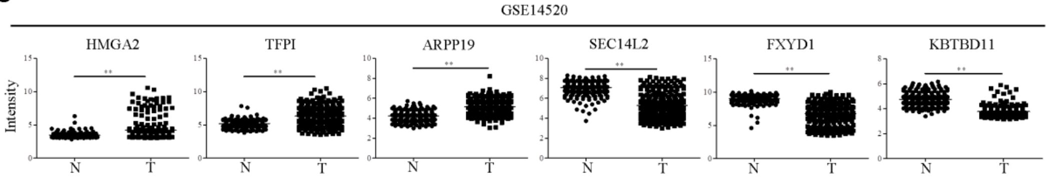

D

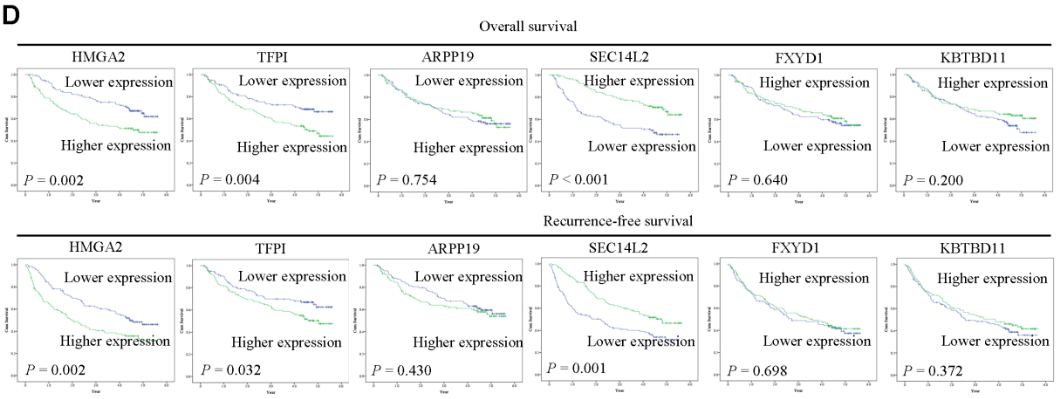

E

**RNA level**	LOC344887	HMGA2	TFPI
LOC344887		R = 0.516<br>*P* < 0.001	R = -0.101<br>*P* = 0.207
HMGA2	R = 0.516<br>*P* < 0.001		R = -0.095<br>*P* = 0.240
TFPI	R = -0.101<br>*P* = 0.207	R = -0.095<br>*P* = 0.240	

Supplementary Figure 4

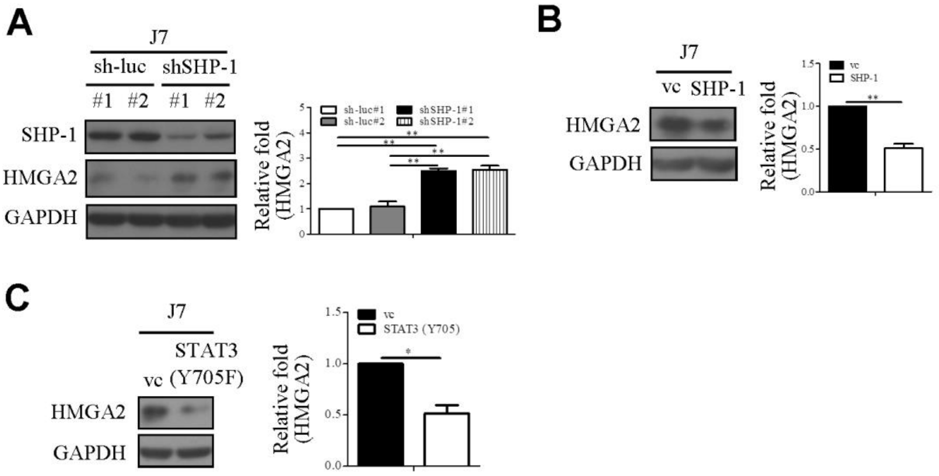

Supplementary Figure 5

A

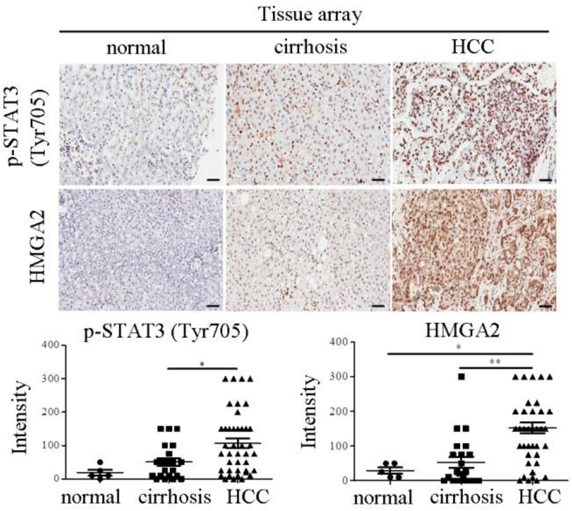

B

	LOC344887 (RNA level)	p-STAT3 Tyr705 (protein level)	HMGA2 (protein level)
LOC344887 (RNA level)		r = 0.310<br>P = 0.048	r = 0.325<br>P = 0.038
p-STAT3 Tyr705 (protein level)	r = 0.310<br>P = 0.048		r = 0.507<br>P < 0.001
HMGA2 (protein level)	r = 0.325<br>P = 0.038	r = 0.507<br>P < 0.001	

Supplementary Figure 6

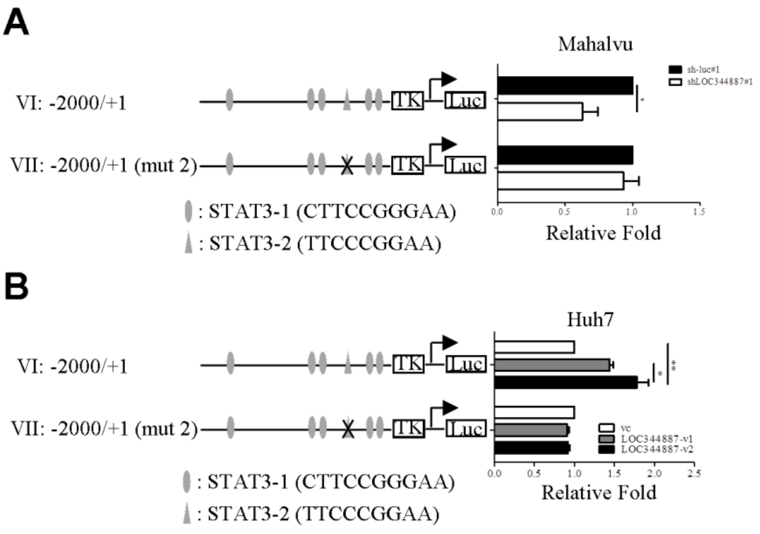

Supplementary Figure 7

Supplementary Table 1. The primers were used in RT-PCR, qRT-PCR, ChIP and shRNA analysis.

RT-PCR
LOC344887 forward primer: 5’- CTCTCTCTTTTCCTGATAAC-3’
LOC344887 reverse primer: 5’- AATATCAGCAACAGAGATAC-3’
18S rRNA forward primer: 5'- CGAGCCGCCTGGATACC-3'
18S rRNA reverse primer: 5'- CCTCAGTTCCGAAAACCAACAA-3’

qRT-PCR
LOC344887 forward primer: 5'-GAGCCTTCAAGGGCATGTAGA-3'
LOC344887 reverse primer: 5'-GAATGTTGTGCCGTAAAGAGGAA-3'
Specific LOC344887-v1 forward primer: 5'-ATAATTCTCACCAGAGAAGAAACA-3'
Specific LOC344887-v1 reverse primer: 5'-TCAGTGGAGTAACAGAACTTTCC-3'
Specific LOC344887-v2 forward primer: 5'-CTGTGGCCAGGGCAATTTT-3'
Specific LOC344887-v2 reverse primer: 5'-CTCCAGGGCATTTGGTCAA-3'
HMGA2 forward primer: 5'- TGGGAGGAGCGAAATCTAA -3'
HMGA2 reverse primer: 5'- GGTGAACTCAAGCCGAAG -3'
TFPI forward primer: 5'- GTGGATGCCTGGGCAATA -3'
TFPI reverse primer: 5'- GGAAACCATTCGGACCATCT -3'
18S rRNA forward primer: 5'- CGAGCCGCCTGGATACC-3'
18S rRNA reverse primer: 5'- CCTCAGTTCCGAAAACCAACAA-3'

ChIP assay
HMGA2 promoter forward-2 primer: 5’-TGTCAGGGGACCTCTCCCAC-3’
HMGA2 promoter reverse-2 primer: 5’-GAGGTCACCCCTGGGCAGTT-3’
GAPDH forward primer: 5’-TACTAGCGGTTTTACGGGCG-3’
GAPDH reverse primer: 5’-TCGAACAGGAGCAGAGAGCGA-3’

shRNA
LOC344887 shRNA-1 forward:
5’-CCGGATACAGCAATATGCTGATGTTCTCGAGAACATCAGCATATTGCTGTATTTTTT-3’
LOC344887 shRNA-1 reverse:
5’-AATTAAAAAATACAGCAATATGCTGATGTTCTCGAGAACATCAGCATATTGCTGTGT-3’
LOC344887 shRNA-2 forward:
5’-CCGGCTCAACCAAGATAAGGAAGTGCTCGAGCACTTCCTTATCTTGGTTGAGTTTTT-3’

LOC344887 shRNA-2 reverse:
5’-AATTAAAAACTCAACCAAGATAAGGAAGTGCTCGAGCACTTCCTTATCTTGGTTGAG-3’

Supplementary Table 2. The antibodies were used in western blot analysis, IHC, PLA and RIP assay.

Antibody	Company	Catalog No.	Dilution/Concentration	Assay
HMGA2	Cell Signaling	#5269	1:1000	WB
			1:100	IHC
p-STAT3 (Tyr705)	Cell Signaling	#9145	1:1000	WB
STAT3	Cell Signaling	#9139	1:1000	WB
SHP-1	Cell Signaling	#26516	1:200	PLA
p-STAT3 (Tyr705)	Merck	05485	1:200	PLA
p-STAT3 (Tyr705)	ABclonal	AP0705	3 μg	RIP
			1:100	IHC
STAT3	ABclonal	A19566	3 μg	RIP
SHP-1	ABclonal	A19111	1:1000	WB,
GAPDH	Santa Cruz	sc-47724	1:8000	WB
